# Ancient and modern genomics of the Ohlone Indigenous population of California

**DOI:** 10.1073/pnas.2111533119

**Published:** 2022-03-21

**Authors:** Alissa L. Severson, Brian F. Byrd, Elizabeth K. Mallott, Amanda C. Owings, Michael DeGiorgio, Alida de Flamingh, Charlene Nijmeh, Monica V. Arellano, Alan Leventhal, Noah A. Rosenberg, Ripan S. Malhi

**Affiliations:** ^a^Department of Genetics, Stanford University, Stanford, CA 94305;; ^b^Far Western Anthropological Research Group, Davis, CA 95618;; ^c^Department of Anthropology, Northwestern University, Evanston, IL 60206;; ^d^Department of Anthropology, University of Illinois, Urbana, IL 61801;; ^e^Carl R. Woese Institute for Genomic Biology, University of Illinois, Urbana, IL 61801;; ^f^Department of Electrical Engineering and Computer Science, Florida Atlantic University, Boca Raton, FL 33431;; ^g^Muwekma Ohlone Tribe of the San Francisco Bay Area, Castro Valley, CA 94546;; ^h^Department of Anthropology, San Jose State University, San Jose, CA 95192;; ^i^Department of Biology, Stanford University, Stanford, CA 94305

**Keywords:** genes and languages, identity by descent, Indigenous population genetics, paleogenomics, Penutian hypothesis

## Abstract

Traditional knowledge, along with archaeological and linguistic evidence, documents that California supports cultural and linguistically diverse Indigenous populations. Studies that have included ancient genomes in this region, however, have focused primarily on broad-scale migration history of the North American continent, with relatively little attention to local population dynamics. Here, in a partnership involving researchers and the Muwekma Ohlone tribe, we analyze genomic data from ancient and present-day individuals from the San Francisco Bay Area in California: 12 ancient individuals dated to 1905 to 1826 and 601 to 184 calibrated years before the present (cal BP) from two archaeological sites and eight present-day members of the Muwekma Ohlone tribe, whose ancestral lands include these two sites. We find that when compared to other ancient and modern individuals throughout the Americas, the 12 ancient individuals from the San Francisco Bay Area cluster with ancient individuals from Southern California. At a finer scale of analysis, we find that the 12 ancient individuals from the San Francisco Bay Area have distinct ancestry from the other groups and that this ancestry has a component of continuity over time with the eight present-day Muwekma Ohlone individuals. These results add to our understanding of Indigenous population history in the San Francisco Bay Area, in California, and in western North America more broadly.

## Significance

California supports a high cultural and linguistic diversity of Indigenous peoples. In a partnership of researchers with the Muwekma Ohlone tribe, we studied genomes of eight present-day tribal members and 12 ancient individuals from two archaeological sites in the San Francisco Bay Area, spanning ∼2,000 y. We find that compared to genomes of Indigenous individuals from throughout the Americas, the 12 ancient individuals are most genetically similar to ancient individuals from Southern California, and that despite spanning a large time period, they share distinctive ancestry. This ancestry is also shared with present-day tribal members, providing evidence of genetic continuity between past and present Indigenous individuals in the region, in contrast to some popular reconstructions based on archaeological and linguistic information.

Among the geographic regions of North America, California is one of the areas with the greatest cultural and linguistic diversity of Indigenous peoples (1–3). With significant coastal and terrestrial ecological productivity, the region supported large precontact populations with the highest population density in North America (4–6). The geographic, cultural, and linguistic complexity of California at European contact contributed to considerable structuring among the Indigenous groups speaking more than 78 mutually unintelligible languages within six major linguistic families (3, 7). Today, California is home to 109 federally recognized sovereign tribal nations and more than 40 non–federally recognized tribal groups.

Considering regions within California, the area surrounding San Francisco Bay in Northern California supported some of the highest regional population densities at the start of European colonization in 1776 (8, 9). Indeed, the 21 Spanish mission locations in California, which were situated in a manner that correlated with Indigenous population density, included five missions located near San Francisco Bay. Population reconstructions using Spanish mission baptismal recruitment records reveal that at contact, more than 15,000 Native Americans from five distinct language groups were residing in sedentary villages within 45 territorial communities (land-controlling autonomous polities) within 20 km of the bay (9–11). Extensive investigation of the region's dense archaeology has produced a trans-Holocene record, revealing that intensive sedentary or semisedentary habitation extends back >5,000 y (11–14).

With a rich regional archaeological record spanning >11,000 y of Indigenous habitation (14), much potential exists for coproduction of knowledge by recovering ancient DNA from Indigenous ancestors and jointly analyzing genetic and archaeological data. To date, however, California and the San Francisco Bay Area have seen little attention in paleogenomic studies. The most detailed study of ancient human genomic data in California has focused on Southern California, considering populations from the Channel Islands (15); additional significant studies of nearby regions have examined Lovelock Cave and Spirit Cave in Nevada (16), as well as the Pacific Northwest (16–18) and Northern Mexico (15, 19).

With generally sparse geographic coverage and relatively few ancient individuals from North America investigated using genomics, studies in the region have often focused on questions concerning initial entry of Indigenous populations into the Americas and broad-scale migration history of early Indigenous groups (15, 16, 19, 20). Studies have often focused on the information revealed about broad-scale population history from a small number of individuals (21–24), with relatively few studies focusing on a specific geographic area and considering multiple sampled individuals (17, 18); another limitation has been the use of genetic sites in mitochondrial DNA rather than genome-wide (25).

In this study, in partnership of researchers with the Muwekma Ohlone tribe of the San Francisco Bay Area, we examine a time transect of a single region of Indigenous habitation, centered on Sunol on the southeast side of San Francisco Bay. The Muwekma Ohlone are one of the descendant communities of Ohlone who originally occupied ∼4.3 million acres from San Francisco to Monterey and from the coast to the upland edge of the Central Valley. The Muwekma Ohlone comprise all the lineages who trace their ancestry through the Bay Area Missions of San Francisco, Santa Clara, and San Jose and who were also members of the historic previously federally recognized Verona Band of Alameda County who resided on the Pleasanton (Alisal), Sunol, Livermore (Del Mocho), and Niles (El Molino) rancherias from post-Spanish mission secularization (1834) to the early 1900s.

We consider human paleogenomic analysis from burials at two adjacent ancestral Ohlone settlements set away from the bay margin near Sunol, one dated to 2,440 to 175 cal BP, the other to 605 to 100 cal BP (26, 27). We also present information derived from living members of the Muwekma Ohlone tribe, considering that their ancestral lands include this locality and noting their strong historical ties to this region in particular that persist to the present day (*SI Appendix*, Table S1). Tribal members trace familial connections to the Sunol region (a 5-mile radius around Sunol includes the historic rancherias listed above and Mission San Jose) over many generations, as reported in interviews with tribal elders and genealogical analysis (28–30). This investigation, considering multiple groups across a range of time periods, provides a case example of joint ancient and modern DNA analysis in a single regional setting.

We combine information from traditional knowledge, genetics, and archaeology to examine the three sets of individuals. First, we investigate the ancient Bay Area individuals in relation to other ancient persons from the Americas, focusing attention on California and surrounding regions. Next, we examine the relationships of individuals between the two sites as well as between the ancient individuals and the modern tribal members, assessing the possibility of genetic continuity among these groups. The analysis reveals that genetic links between ancient and modern populations are evident despite the extreme disruption to the Ohlone that occurred during Spanish occupation and subsequent incorporation of the region into Mexico and then the United States—including forced migration to the missions and reductions in lifespan due to new diseases and the conditions of mission life (9, 31–33). The broader genetic context inferred for the three sets of individuals deepens understanding of Indigenous population history of California and the San Francisco Bay Area.

## Results

### Community Engagement.

Large-scale infrastructure construction led to the excavation of two Ohlone villages, *Síi Túupentak* (CA-ALA-565/H) and *Rummey Ta Kuččuwiš Tiprectak* (CA-ALA-704/H), in Sunol, CA (see *Archaeological Investigation*). Far Western Anthropological Research Group completed the excavations in partnership with the Tribe, with community members participating in all aspects of fieldwork as well as being the primary excavators of all burials.

The genomics section of the project began in 2016 after Tribal Council requested and approved a study design for the endeavor. The study design included community-based methods (34–36). After the project began, members of the research team visited the sites and met with Tribal Council and community members multiple times to have discussions on the latest results of the project, safeguards to be used for the data generated in the project, and thoughts on paths forward for the study. During the time period of the COVID-19 pandemic, the research team met virtually with Tribal Council and community members. Prior to the start of the project, members of the Tribe attended the Summer Internship for INdigenous Peoples in Genomics program in 2011 and 2013 to learn about the latest genomic analyses and about topics in the ethical, legal, and social implications of genomics research with Indigenous communities. Importantly, members of the Muwekma Ohlone tribe contributed to manuscripts and news stories published or disseminated about the project.

### Archaeological Investigation.

**Fig. 1.**
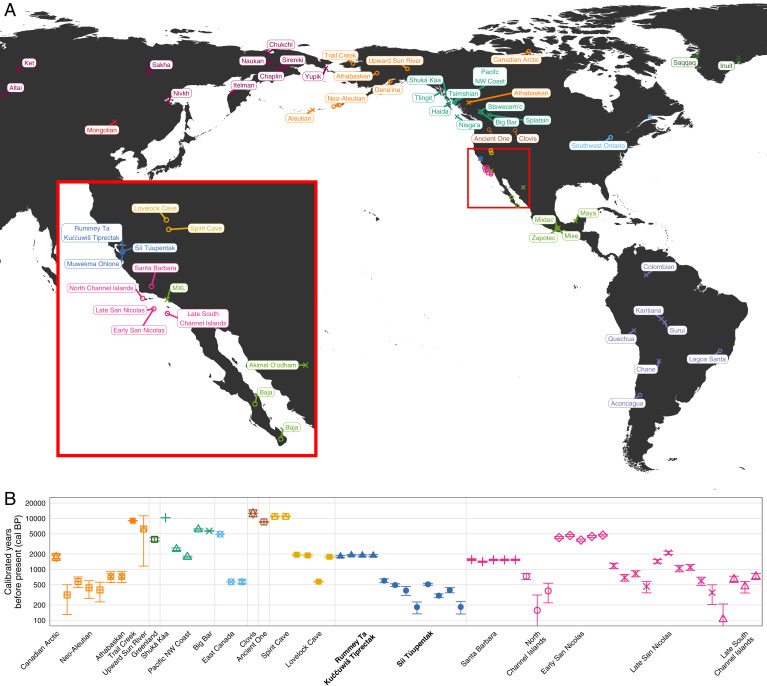
Identifiers for ancient and present-day individuals used in this study. (*A*) Map of ancient and present-day individuals, colored by regional grouping. The inset shows the new individuals from the San Francisco Bay Area (blue) and the surrounding groups. (*B*) Dates of ancient individuals included in the dataset.

*Síi Túupentak* (CA-ALA-565/H) and *Rummey Ta Kuččuwiš Tiprectak* (CA-ALA-704/H) are both ancestral Native American Ohlone settlements situated in an open valley of the southeast San Francisco Bay region, central California, USA (Fig. 1). Modern development for large-scale infrastructure construction necessitated that substantive archaeological excavations be conducted at both sites. Archaeological mitigation of construction impacts to these archaeological sites, including the identification, excavation, analysis and reporting of human remains, strictly conformed to all state and local laws and regulations.

The Muwekma Ohlone tribe was appointed Most Likely Descendant Tribe for the project by the state and, in 2015, prior to the development of the research design for archaeological investigations, recommended detailed analysis, including paleogenomics, of all ancestral remains that may be encountered. All research designs, analytical studies of ancestral remains, and reports were reviewed and approved by tribal leadership, and the Tribe partnered with the research team to conduct these investigations.

*Síi Túupentak* (“*Place of the Water Round House Site*”) is a large (2.8 ha/6.9 acres), intensively occupied sedentary village consisting of a thick deposit of cultural material creating a low, anthropogenic mound, along with an associated cemetery (27). Archaeological investigation of 6.2% of the site recovered a wide range of cultural remains, including more than 13,000 artifacts, numerous food remains, 36 features, and 66 burials comprising 76 individuals. The site dates from 605 to 100 cal BP (1345 to 1850 CE), based on 129 radiocarbon dates from features, burials, and generalized site deposits. The site was founded prior to European contact and continued to be inhabited during early European coastal exploration starting in 1542 CE and through the region’s Spanish colonization, until the inhabitants were forced into the Spanish mission compounds (1776 to 1807). The site was also briefly reoccupied in the 1830s after the collapse of the Spanish empire. The eight individuals in this study include six females and two males of varied ages at death, and they span the full time range of occupation.

The nearby site of *Rummey Ta Kuččuwiš Tiprectak* (“*Place of the Stream of the Lagoon Site*”) has a similar size but is a multicomponent settlement including a precolonial Indigenous occupation and a subsequent colonial Mexican and Early American period ranch complex in use from 1839 CE to the early 1900s. The Native American component of the site includes artifactual and other debris, 44 features, and 25 burials comprising 29 individuals. This component was inhabited from 2,440 to 175 cal BP (490 BCE to 1775 CE), based on 60 radiocarbon dates from generalized site deposits, features, and burials (26). The settlement was most intensive between 2,440 to 1,610 cal BP (88% of dates fall in this time span). The six individuals for which genomic analysis was attempted include four females and one male, including two children and three adults, and date from 1,905 to 1,785 cal BP.

### Genetic Dataset.

We whole-genome sequenced 12 ancient individuals from two archaeological sites in the San Francisco Bay Area to a depth of 0.06 to 7.8× and mean 2.4×, after excluding two samples from the *Rummey Ta Kuččuwiš Tiprectak* site without sufficient genetic material (*SI Appendix*, Table S2 and Figs. S1–S3). Individuals from *Síi Túupentak* dated to 601 to 184 cal BP, and individuals from *Rummey Ta Kuččuwiš Tiprectak* dated to 1905 to 1826 cal BP. We also whole-genome sequenced eight present-day members of the Muwekma Ohlone tribe to high coverage, ranging from 18 to 25×. We assembled a dataset of relevant previously published samples. This dataset included 291 individuals from Asia, Europe, North America, and South America; it contained 68 ancient individuals and 223 modern individuals (*SI Appendix*, Table S3; Fig. 1*A*). After merging the new and previously published individuals, the dataset we analyzed contains 311 individuals, 80 ancient individuals and 231 present-day individuals, typed for 474,317 single-nucleotide polymorphisms (SNPs; see *SI Appendix*, *Methods*).

Radiocarbon dates of the 12 newly sampled ancient individuals and those available for the previously published individuals are shown in Fig. 1*B*. Focusing on the ancient individuals from Nevada and California, we see that the dates fall into approximately three periods. The oldest group, from >4,000 cal BP, includes the individuals from Spirit Cave and those labeled Early San Nicolas. An intermediate group with ages between 2,000 to 1,500 cal BP includes the Lovelock Cave, *Rummey Ta Kuččuwiš Tiprectak*, and Santa Barbara groups. The most recent set includes individuals from *Síi Túupentak*, North Channel Islands (in this study, San Miguel and Santa Cruz), Late San Nicolas, and South Channel Islands (San Clemente and Santa Catalina), mostly with dates <1,000 cal BP.

### Overview of Data Analysis.

Using the sample of 311 present-day and ancient individuals, we performed principal components analysis (PCA) and model-based clustering analysis to identify genetic relationships among previously reported individuals, the newly sampled ancient individuals, and the present-day Muwekma Ohlone individuals. We then restricted attention to a subset of 165 individuals with ancestry relevant to the new individuals, and repeated the analysis, also analyzing identity-by-state (IBS) segment sharing (*SI Appendix*, Table S4). Whereas the PCA and model-based clustering analyses use the genotypes of the 474,317 SNPs directly, in order to identify IBS segments, we imputed genotypes from the ancient samples across the whole genome (*Materials and Methods*). In interpreting the results of the various analyses, we considered the relationships of the 12 newly sampled ancient individuals and eight present-day Muwekma Ohlone individuals to other individuals, as well as to each other.

### The San Francisco Bay Area Individuals in the Context of Native American Genetic Diversity.

**Fig. 2.**
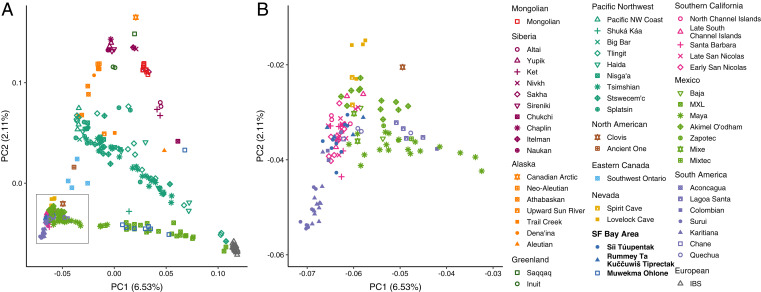
PCA of all ancient and present-day individuals. (*A*) PCA of 311 individuals in the full dataset, including 231 modern and 80 ancient individuals. (*B*) An enlarged view of the cluster in the *Bottom Left Corner* of *A*.

First, using PCA and unsupervised model-based clustering, we explore the relationship between the San Francisco Bay Area individuals and previously published ancient and present-day individuals from surrounding regions. Fig. 2*A* shows a PCA plot of 311 individuals. European individuals cluster in the lower right corner of the plot, and the northernmost populations from Siberia, Alaska, and Greenland appear at the top of the figure. The lower left corner contains a cluster of individuals from California, Nevada, Mexico, and Central and South America.

Clines are visible between these three corners of the plot. Three clines connect the left edge of the plot to the right corner of Europeans. Several Siberian individuals are placed along the upper right edge, a line of Pacific Northwest individuals connects the center of the left edge to the right corner, and a line of individuals from Mexico connects the lower left corner to the corner containing Europeans. These clines appear to reflect varying European admixture that aligns with principal component 1 (PC1). Present-day members of the Muwekma Ohlone tribe, who have a known history of admixture with European Americans, Mexicans, and Mexican Americans, fall along the lower edge, with variable values for PC1.

Focusing on the cluster of individuals from California, Nevada, Mexico, and South America, Fig. 2*B* enlarges the lower left corner of Fig. 2*A*. In the enlarged view, individuals from South America appear in the bottom left corner, anchoring a south-to-north cline along PC2. At the top of Fig. 2*B*, the individuals from Lovelock Cave in Nevada, who are close in age to those from the *Rummey Ta Kuččuwiš Tiprectak* and Santa Barbara sites (Fig. 1*B*), fall above the main cluster. The ancient individuals from the San Francisco Bay Area cluster with the ancient individuals from Southern California along the left edge of Fig. 2*B*.

**Fig. 3.**
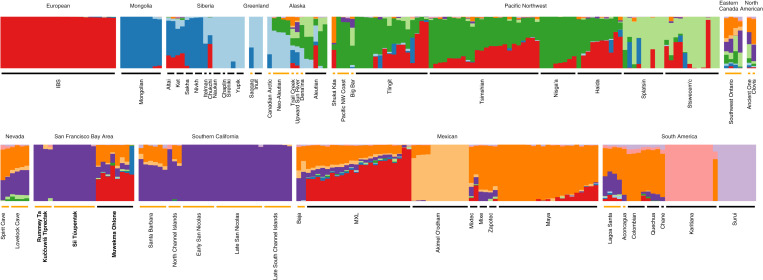
Model-based clustering of all 311 ancient and present-day individuals, with *K* = 10 clusters. The results represent a summary of 10 independent runs of unsupervised clustering. Each of the 10 clusters is represented by a color, and each individual is represented by a vertical bar. To aid interpretation, clustering results from a unified analysis are depicted over two rows. Ancient individuals are denoted by an orange horizontal line below the plot, and present-day individuals are denoted by a black horizontal line.

Model-based unsupervised clustering for *K* = 10 clusters, performed using NGSadmix, appears in Fig. 3. From Asia to South America, we first observe a cluster that appears largely in Mongolia and Siberia (dark blue) and a cluster that appears in Siberia, Greenland, and Alaska (light blue). Two clusters appear primarily in the Pacific Northwest and Alaska, with one centered on Stswecem’c and Splatsin (light green) and the other appearing in most other populations from the region (dark green). A sample of Europeans is assigned to a single cluster, which is seen in many populations in the plot (red). Among the remaining five clusters, three are centered on specific populations: Akimel O’odham (formerly termed Pima; light orange), Karitiana (pink), and Surui (light purple). One is centered on native populations of Mexico and South America (dark orange).

The ancient individuals from the San Francisco Bay Area and Southern California both have majority membership in the same component (purple). As in the PCA, these two groups cluster together. We also observe, as seen by Scheib et al. (15), that the ancient individuals from Southern California separate into two groups: Individuals from San Nicolas and the Southern Channel Islands have membership primarily in a single component (purple), whereas individuals from Santa Barbara and the North Channel Islands have more substantial membership in a second component as well (orange). As was seen by Moreno-Mayar et al. (16), we find that the individuals from Lovelock Cave in Nevada have noticeable membership in a component shared with those from the Pacific Northwest (light green, dark green), a similar signal to their separation in the PCA plot in Fig. 2*B*. The present-day Muwekma Ohlone are known to have European, Mexican, and Ohlone genealogical ancestors, consistent with the appearance of the red, orange, and purple components observed in these individuals.

### Population Structure Within Western North American Populations.

Next, we consider a subset of 165 individuals to more closely examine population structure within western North America. For this subset, we perform PCA, model-based clustering, and analysis of IBS segment sharing.

**Fig. 4.**
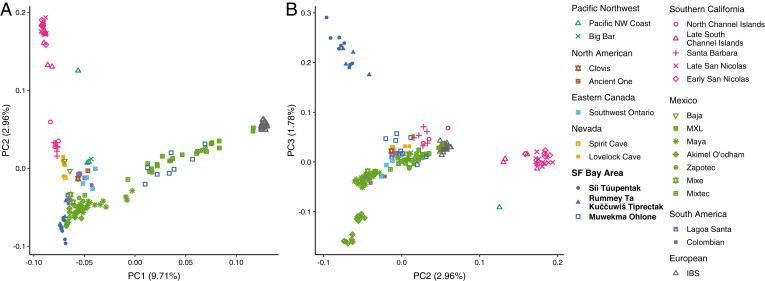
PCA of a subset of ancient and present-day individuals, considering 165 samples with ancestry relevant to newly sampled individuals from the San Francisco Bay Area. (*A*) PCs 1 and 2. (*B*) PCs 2 and 3.

Fig. 4*A* shows the first two principal components. The ancient individuals from San Nicolas and the South Channel Islands cluster are in the top left corner, with the remaining Southern California individuals from Santa Barbara and the North Channel Islands appearing below them along the left side. The European individuals cluster on the right side. Most remaining individuals cluster in the bottom left corner, including those from *Rummey Ta Kuččuwiš Tiprectak* and *Síi Túupentak*. Muwekma Ohlone and MXL (Mexican in Los Angeles) individuals fall along a cline connecting the lower left corner to the cluster containing Europeans, the same cline observed in Fig. 2*A*.

We plot PC2 with PC3 in Fig. 4*B*. In this plot, the individuals from *Rummey Ta Kuččuwiš Tiprectak* and *Síi Túupentak* separate from the large cluster that appeared in the lower left corner of Fig. 4*A*. In the top left corner, the individuals from *Rummey Ta Kuččuwiš Tiprectak* and *Síi Túupentak* cluster together. Populations placed near these individuals in Fig. 4*A*, including several Indigenous populations from Mexico, appear in the center and lower left corner.

**Fig. 5.**
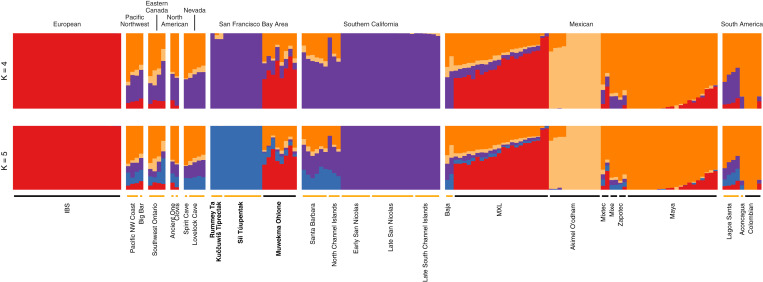
Model-based clustering of a subset of ancient and present-day individuals, considering 165 samples with ancestry relevant to newly sampled individuals from the San Francisco Bay Area. Separately for *K* = 4 and *K* = 5, the results represent a summary of 10 independent runs of unsupervised clustering. Coloring is the same as described in Fig. 3.

Inferences with unsupervised model-based clustering for *K* = 4 and 5 appear in Fig. 5. At *K* = 4, we observe four clusters that are largely similar to four of the clusters seen in the *K* = 10 analysis shown for the larger dataset in Fig. 3. The European individuals are placed in one cluster (red), the Akimel O’odham individuals are assigned primarily to a second cluster (light orange), a third cluster is centered on individuals from Mexico (dark orange), and a fourth is centered on the ancient individuals from California (purple).

Increasing *K* to 5 splits the purple cluster into two, with the purple cluster remaining centered on the individuals from Southern California and the new blue cluster centered on the ancient individuals from the San Francisco Bay Area. A small amount of membership is seen in this blue cluster in other populations, including the individuals from Santa Barbara and the North Channel Islands, the Spirit Cave and Lovelock Cave samples, the North American samples, the Lagoa Santa sample from South America, and the modern Muwekma Ohlone.

**Fig. 6.**
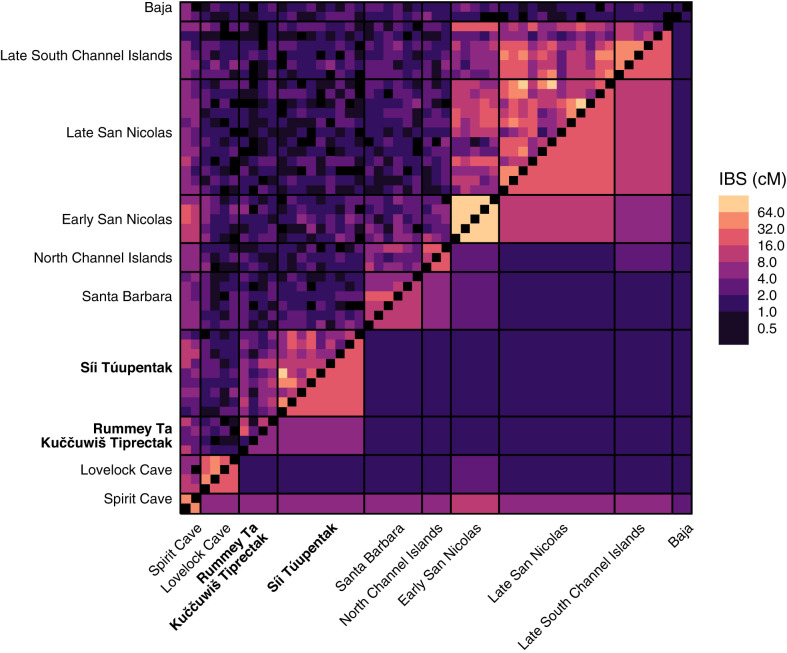
Total pairwise IBS segment sharing for 53 ancient individuals from Nevada, California, and the Baja peninsula. The upper triangle of the matrix shows the total length of segments shared for pairs of individuals. The lower triangle shows mean pairwise values. In the triangularly shaped regions incident to the diagonal, the mean is taken across pairs within a population; rectangles off the diagonal show means across pairs, one from one population and one from another population.

To further understand population structure in western North America, we evaluate IBS genomic sharing between pairs of individuals, focusing on 53 ancient individuals from Nevada, California, and Mexico and employing genome-wide imputed genotypes (Fig. 6). The individuals from the oldest site, Spirit Cave in Nevada, share segments broadly, potentially reflecting ancestral haplotype sharing with many more recent individuals because of their increased ages. To some extent, a similar pattern is seen for individuals from the next oldest site, Early San Nicolas.

The highest levels of IBS sharing occur along the diagonal between individuals from the same population. The analysis suggests four clusters—Nevada, San Francisco Bay Area, North Channel Islands together with Santa Barbara, and South Channel Islands—for which pairs within a cluster possess elevated IBS sharing relative to pairs from distinct clusters. Segment sharing decreases for pairs from distinct clusters, with the exception of the sharing between individuals from the North Channel Islands and the Late South Channel Islands, who are close in age.

The clustered pattern of IBS sharing mirrors observations seen in Figs. 4 and 5. Because the highest levels of sharing occur within these population clusters and because the individuals in a cluster have a range of ages, the IBS sharing within each cluster suggests population continuity over space and over time, in the sense that subsequent populations possess ancestry in prior populations. Focusing on the San Francisco Bay Area, the elevated sharing between the individuals from the older *Rummey Ta Kuččuwiš Tiprectak* site and the more recent *Síi Túupentak* site and the relatively low sharing of these individuals to others suggest a notable level of genetic continuity in time between the two sites and that at both of the time periods they represent, their populations possessed distinct ancestry from contemporaneous individuals in Nevada and Southern California.

### Present-day Muwekma Ohlone and Ancient *Rummey Ta Kuččuwiš Tiprectak* and *Síi Túupentak* Individuals.

Present-day members of the Muwekma Ohlone tribe are known to possess European, Mexican, and Ohlone genealogical ancestors, and we observe this history of admixture in many of our analyses. In Figs. 2*A* and 4*A*, the Muwekma Ohlone lie along a cline on PC1, reflecting European and Mexican admixture. In Figs. 3 and 5, the largest cluster memberships for the Muwekma Ohlone appear in the cluster centered on the European individuals (red) and the cluster centered on Indigenous individuals from Mexico (dark orange).

Despite this signal of admixture, the analyses consistently suggest shared ancestry between the Muwekma Ohlone and the individuals from the *Rummey Ta Kuččuwiš Tiprectak* and *Síi Túupentak* sites. In Fig. 3 and in the analysis with *K* = 4 in Fig. 5, the Muwekma Ohlone share membership with the ancient individuals from California, both those from the San Francisco Bay Area and those from Southern California (purple). In Fig. 5, at *K* = 5, we also see that the cluster centered on the individuals from *Rummey Ta Kuččuwiš Tiprectak* and *Síi Túupentak* is visible in the Muwekma Ohlone (blue).

**Fig. 7.**
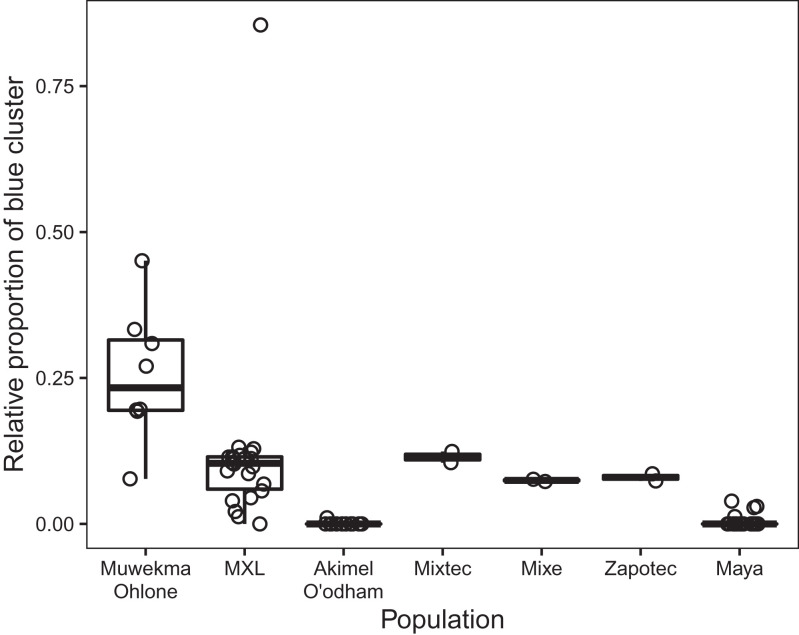
Membership in the blue cluster in the *K* = 5 cluster analysis in Fig. 5, divided by one minus membership in the red cluster, for present-day populations from California and Mexico. For each individual, the proportion is calculated as the membership fraction in the blue cluster divided by the total fraction of membership in the blue, purple, light orange, and dark orange clusters. Individual values and boxplots are shown.

By excluding membership corresponding to European admixture, we can compare the shared membership that the Muwekma Ohlone possess with the cluster centered on *Rummey Ta Kuččuwiš Tiprectak* and *Síi Túupentak* to corresponding shared membership that other modern populations possess with this cluster. In Fig. 7, for various modern populations, we consider the relative proportion that appears in the blue component in the *K* = 5 plot in Fig. 5 in modern individuals, as a fraction of total membership excluding the red component centered on the European individuals. This analysis reveals that the Muwekma Ohlone possess a larger relative proportion of the blue component than do other populations; Mann-Whitney tests for the eight Muwekma Ohlone produce *P* = 5.6 × 10^−4^ for a comparison with 22 MXL individuals, *P* = 3.0 × 10^−5^ with 12 Akimel O’odham individuals, and *P* = 4.0 × 10^−6^ with 21 Maya individuals (with small sample sizes of two individuals each, *P =* 0.09 with Mixtec, *P* = 0.02 with Mixe, and *P* = 0.04 with Zapotec). Hence, despite the admixture history of the Muwekma Ohlone, so that the population possesses multiple membership components, one membership component shared between the Muwekma Ohlone and the ancient individuals from the *Rummey Ta Kuččuwiš Tiprectak* and *Síi Túupentak* sites—a component suggestive of a partial shared ancestry—can be observed. This sharing between the present-day and ancient individuals is further supported in additional tests using the *f*_4_ statistic, by which greater similarity is observed between the Muwekma Ohlone and the *Rummey Ta Kuččuwiš Tiprectak* and *Síi Túupentak* sites than between the Muwekma Ohlone and ancient individuals from surrounding regions (*SI Appendix*, Table S5).

## Discussion

In this study, we sequenced genomes of 12 ancient individuals from two archaeological sites in the San Francisco Bay Area and eight present-day members of the Muwekma Ohlone tribe. To study population structure within California and western North America more broadly, we compared these individuals to previously published genomes of ancient and present-day Indigenous individuals. We also compared the 12 ancient individuals and eight modern individuals from the San Francisco Bay Area.

### Continuity of Ancient and Modern Populations in the San Francisco Bay Area.

We first performed analyses of the newly sampled ancient individual genomes with a broad sample containing individuals from North America, South America, Europe, and Siberia. In these analyses, the ancient individuals from the *Rummey Ta Kuččuwiš Tiprectak* and *Síi Túupentak* sites clustered most closely with the ancient individuals from Southern California. Using PCA, the individuals from these groups overlap (Fig. 2), and with model-based clustering, we see that a shared cluster is centered on them (Fig. 3, purple).

Next, we focused our analysis on a subset of populations with ancestry relevant to the newly sequenced ancient individuals. In finer-scale analysis, the ancient individuals from the San Francisco Bay Area and Southern California, who cluster together in the larger dataset, are split into separate clusters. With PCA, the individuals from *Rummey Ta Kuččuwiš Tiprectak* and *Síi Túupentak* cluster together (Fig. 4), and with model-based clustering, at *K* = 5, a cluster is centered on the ancient individuals from the San Francisco Bay Area (Fig. 5, blue). In an analysis of IBS sharing, we find elevated sharing among the ancient San Francisco Bay Area individuals from the two archaeological sites relative to the sharing with individuals from Mexico, Nevada, and Southern California.

Finally, we considered the relationship between the ancient individuals from *Rummey Ta Kuččuwiš Tiprectak* and *Síi Túupentak* and their relationship to the present-day Muwekma Ohlone. Although the present-day individuals also possess recent European and Mexican ancestry, we find that they also share ancestry with the ancient individuals. In particular, considering fractions of individual genomes estimated to have Indigenous ancestry, we found in Fig. 7 that the Muwekma Ohlone share a relatively high proportion of a cluster shared with the ancient individuals from the San Francisco Bay Area (blue cluster in Fig. 5).

The shared ancestry components provide support for genetic continuity between the individuals from the *Rummey Ta Kuččuwiš Tiprectak* and *Síi Túupentak* archaeological sites and between the two sites and the present-day Muwekma Ohlone. This continuity, in the sense of a possible genealogical descent relationship connecting the more ancient and more recent populations, would then extend from the *Rummey Ta Kuččuwiš Tiprectak* individuals, dated to 1905 to 1826 cal BP, through the *Síi Túupentak* individuals, who date to 601 to 184 cal BP, to current tribal members. The two archaeological sites represent substantially longer time periods than the dates associated with the particular individuals sampled; *Rummey Ta Kuččuwiš Tiprectak* was inhabited 2440 to 175 cal BP, most actively during 2440 to 1610 cal BP, and *Síi Túupentak* spans 605 to 100 cal BP. The genetic connections between the two archaeological sites and between the sites and the present-day Muwekma Ohlone individuals suggest that the present-day Muwekma Ohlone share continuity with peoples who have inhabited the San Francisco Bay Area for at least two millennia, since the genetic sampling period for *Rummey Ta Kuččuwiš Tiprecta*k, 1905 to 1826 cal BP, and potentially to the earliest dates of the site, around 2440 cal BP. These results suggest that models in which ancestral Ohlone populations are posited to have migrated to the region 1,500 to 1,000 y ago (3, 37, 38) provide underestimates of the continuity of the population. They are compatible with reconstructions that posit Ohlone population continuity in this portion of the San Francisco Bay Area extending back to 2,500 y ago or possibly earlier (39–41).

We note that the population continuity we have observed between the archaeological sites and the current Muwekma Ohlone takes the form of a continuity of genetic ancestry components and a noteworthy sharing of genomic segments. This form of genetic continuity does not provide formal evidence that the modern individuals are directly descended from the individuals studied from these archaeological sites, but it is compatible with a view that the modern population is descended from those in the archaeological sites or from genetically similar contemporaneous populations. That this continuity is detectable is perhaps surprising, considering the extreme disruption and increase in deaths of the Ohlone caused by Spanish occupation. Mission records document substantial intermixture with neighboring non-Ohlone groups that began after other tribal groups (notably Coast Miwok, Bay Miwok, Plains Miwok, and Yokuts) from neighboring areas were brought to the same missions because of the rapid decline of Bay Area Ohlone mission populations (9, 42). As a result, for example, some descendants of marriages between Ohlone and non-Ohlone individuals identified culturally as Ohlone, spoke the language, and maintained key cultural traditions (28, 30, 43). Genetic continuity with the archaeological sites is detectable despite this intermixture of Indigenous populations from locations relatively distant from the sites.

### Interpretations in Relation to the Penutian Language Family.

Attempts to explain the complex mosaic of California languages and language families at European contact have given primacy to historical linguistic reconstructions that posit successive precontact migrations and displacements of various language groups and approximate timings of language divergence within families (44–46). Archaeologists have then looked for changes in the precontact archaeological record that would test these models. As a result, precontact California history is often framed as possessing linguistic and archaeological cultural concordance (40, 47). With respect to the San Francisco Bay Area, this view holds that speakers of Hokan languages initially occupied central California. Subsequently, Hokan speakers were then pushed to geographic peripheries by Penutian speakers entering California in a series of migrations and inhabiting the Central Valley and Bay Area (3, 13, 40, 48). Proto-Penutian speakers in California are hypothesized to have originated in the Great Basin or possibly on the Columbian Plateau. This hypothesis has been based on historical linguistic reconstructions, archaeological investigations, and recent mitochondrial DNA research (3, 40, 49)—notably including similarities in material culture (projectile point types, stone pipes, extensive bone tool industry with distinctive types, and basketry techniques) between the Lovelock Culture of western Nevada and the appearance of the Windmiller Pattern in central California during the Late Holocene (40, 48, 50). The Ohlone language falls within the geographically extensive Penutian language family, most closely related to the neighboring Miwok and Yokuts languages (44–48).

The four ancient Lovelock Cave individuals are clustered to some extent with ancient individuals from the San Francisco Bay Area and Southern California (Fig. 2). They also share two ancestry clusters with ancient and modern individuals from the Pacific Northwest (Fig. 3, light green, dark green). Four ancient Pacific Northwest Coast individuals, along with the ancient Big Bar individual also from the Pacific Northwest, possess a small amount of membership in a cluster shared with the ancient individuals from Nevada and California (Fig. 3, purple). These patterns are compatible with a view that the Lovelock Cave individuals share similarities with Penutian groups that spread both into the Pacific Northwest and into California (48). In this view, the shared ancestry component could represent a signature of a spread of the Penutian languages, with the Lovelock Cave individuals and the Pacific Northwest Coast and Big Bar individuals both descended from ancestors in the Great Basin region (Fig. 3, purple).

Despite this similarity to the ancient individuals from Lovelock Cave, both the ancient San Francisco Bay Area individuals and the present-day Muwekma Ohlone individuals clustered more closely with ancient individuals from Southern California, where the Penutian language family is absent, than with the (possibly Penutian-speaking) Lovelock Cave individuals associated with Lovelock Culture. Because our analyses do not cluster individuals associated with putative regions of Penutian speakers together (e.g., Lovelock Cave, Pacific Northwest, San Francisco Bay Area), we can conclude that if Penutian languages did spread from the Great Basin into California, then either the spread might have involved linguistic rather than demic diffusion or a shared genetic signal of an initial migration has been eroded by subsequent demographic processes. In both scenarios, genetic and linguistic histories in California are not coupled, so that a history of the spread of cultures in the region is unlikely to always align with the spread of languages. This perspective is consistent with the challenges archaeologists have noted in trying to link historical linguistic models of migrations of populations speaking specific languages with clear changes in the archaeological record, resulting in widely divergent suggestions for the timing of these migration events (13, 14, 51).

We note that in Southern California, we observed a consistent separation of South Channel Islands and San Nicolas individuals from individuals from the North Channel Islands and Santa Barbara, amplifying a pattern visible in figure S11 in Scheib et al. of ref. 15. The ancient individuals from Santa Barbara and the North Channel Islands cluster with the ancient San Francisco Bay Area samples, separating from the individuals from the South Channel Islands, including the individuals from San Nicolas Island. This separation accords with a language boundary at the time of European contact: Individuals from Santa Barbara and the North Channel Islands spoke Chumash languages (considered either part of the Hokan group or an ancient linguistic isolate), whereas individuals from the South Channel Islands (plus San Nicolas) spoke Takic languages of the Uto-Aztecan group (45, 46). Takic language speakers are hypothesized to have migrated from the Great Basin into Southern California during the last 5,000 y, with uncertain timing of their arrival on the coast and the South Channel Islands (1, 40, 45, 52). The genetic clustering of Early San Nicolas Island individuals (dated from 5,000 to 4,000 cal BP) with Late San Nicolas Island individuals (dated to 2,000 cal BP or later) but separate from individuals from the North Channel Islands and Santa Barbara suggests population continuity on San Nicolas during this time span and is compatible with the reconstruction that posits an early arrival of Takic language speakers on San Nicolas.

### Methodological Considerations.

Because of the poor read quality and low sequencing depth for ancient samples, analysis of ancient DNA has primarily made use of haploid genomes in which the haplotype phase has been lost. However, the augmentation of ancient samples with modern reference genomes is increasingly making it possible to perform genotype imputation and haplotype phasing in ancient samples (53). Previous studies have used imputed diploid genotypes from ancient individuals to study demographic history and estimate phenotypes in ancient individuals (54–58). Our work is one of relatively few studies that use imputed genotypes in ancient samples to evaluate haplotype sharing within and between ancient and present-day individuals (55–57).

In this study, we encountered a scenario in which a modern population of interest to examine for genetic continuity with ancient populations possesses admixture components that are not informative about the relationships of interest. Such scenarios can be addressed by performing analyses that disregard those admixture components. In our scenario, we sought to discern, within the component of genomic membership not assigned to European admixture, relative contributions of clusters associated with different Indigenous populations (Fig. 7). The signature of similarity between present-day Muwekma Ohlone and a cluster with considerable membership in the ancient San Francisco Bay Area samples and smaller signatures of other modern populations with this cluster suggests the potential of the approach in other comparisons of ancient populations to modern admixed populations.

Many ancient DNA studies in the Americas, and particularly those involving individuals from North America, have studied large-scale processes such as the initial peopling of the continents (19, 21, 22, 24) or subsequent major migration events (15, 16). As a result, enough ancient individuals have been sequenced to provide reference data for studies that focus on ancient genomics of a specific region, such as the Pacific Northwest (18) or the Caribbean (59, 60). Our study of ancient and present-day individuals from the San Francisco Bay Area contributes an example of the use of regionally focused ancient genomics to demonstrate how analysis of ancient and modern individuals can reveal changes in local population structure over time.

An important component of this study has been its community engagement process and coproduction of knowledge as part of increasing interest in partnerships between researchers and Indigenous communities to conduct genetic research (34, 36, 61)—including genetic research that involves Indigenous ancestors (35, 62). A distinctive feature in this case has been the participation of a tribal group in the initiative to pursue the project, in the selection of research questions, in archaeological excavation and ancient genomics involving sites in their historical lands, and in present-day genomic analysis with current tribal members. Hence, in addition to its scientific conclusions, the study provides a contribution to advancing community engagement models in Indigenous genomics. The study reaffirms the Muwekma Ohlone’s deep-time ties to the area, providing evidence that disagrees with linguistic and archaeological reconstructions positing that the Ohlone are late migrants to the region (37, 38). The results have also generated interest from tribal leadership in carrying out similar genomic investigations on ancestral remains from older sites in order to better document and understand the time depth of Ohlone population-genetic continuity in the San Francisco Bay region.

## Materials and Methods

### Ethics Approvals.

The study proceeded with significant community engagement at all stages (*Community Engagement*), under Institutional Review Board protocol no. 10538 from the University of Illinois at Urbana–Champaign, and it included informed consent from present-day members of the Muwekma Ohlone tribe. In addition, the Muwekma Ohlone Tribal Council also approved the study, including the genomic analysis of community members and ancestral remains. The Tribal Council was consulted on the results and approved the manuscript for disseminating the study.

### Principal Components Analysis.

We performed PCA with both the full set of 311 and the subset of 165 individuals, employing all 474,317 SNPs. For both datasets, we first estimated the covariance matrix of individual genotype vectors from genotype likelihoods (*SI Appendix*, *Methods*). We then used the eigen function in R to calculate eigenvectors, corresponding to principal components, and eigenvalues.

### Model-Based Clustering.

We used NGSadmix (63) to perform unsupervised model-based clustering on genotype likelihoods from the 85,659 SNPs that remained after LD pruning. For each tested number of clusters *K*, we performed the clustering 10 independent times, running NGSadmix with parameters -minMaf 0.05, -maxiter 10,000, and -tol 0.000001. We also included the parameter -minInd 35 for the full dataset of 311 individuals and -minInd 15 for the subset of 165 individuals. To evaluate the clustering solutions inferred by NGSadmix, we ran CLUMPP (64) with parameters DATATYPE 0, M 2, W 0, S 2, and GREEDY_OPTION 2, and REPEATS 1000. Next, following Verdu et al. (65), we clustered the runs based on pairwise G′ values greater than 0.9. For the majority cluster of each *K* value, which contained the most runs, we reran CLUMPP with the same parameters to produce an averaged clustering solution for display in figures. Preferred choices for the value of *K* were obtained by use of evalAdmix (ref. 66; *SI Appendix*, *Methods* and Fig. S4).

### IBS Segment Sharing.

We identified IBS segments between pairs of samples in four steps (*SI Appendix*, Fig. S5). First, we estimated genotype likelihoods in the ancient and modern samples with ANGSD (67). Second, we phased and imputed genotypes from the genotype likelihoods with GLIMPSE (68). Third, we called IBS segments from the phased genotypes with hap-IBD (69). Fourth, in modern admixed individuals, we performed local ancestry assignment and identified IBS segments that lie on the Indigenous background, considering comparisons between modern samples and other modern samples, and between modern samples and ancient samples. This pipeline generated a list of IBS segments shared between ancient and modern individuals, restricting attention to the Indigenous-origin segments of the modern genomes. Further details appear in the *SI Appendix*, *Methods* and Fig. S6.

## Supplementary Material

Supplementary File

## Data Availability

Genomic data from previous studies have been obtained from public sources, as described in the supplementary material. The Muwekma Ohlone Tribe will review requests for genomic data on tribal members and associated archaeological sites before access can be granted. Please send requests to the corresponding authors.
