# Supplementary material for: Ancient and modern genomics of the Ohlone Indigenous population of California
Source: Proc Natl Acad Sci U S A. 2022 Mar 21;119(13):e2111533119. doi: 10.1073/pnas.2111533119 (PMC9060455; doi:10.1073/pnas.2111533119)
Supplement: Supplementary File [file pnas.2111533119.sapp.pdf]

PNAS
www.pnas.org

Supplementary Information for

Ancient and modern genomics of the Ohlone Indigenous population of California

Alissa L. Severson, Brian F. Byrd, Elizabeth K. Mallott, Amanda C. Owings, Michael DeGiorgio, Alida de Flamingh, Charlene Nijmeh, Monica V. Arellano, Alan Leventhal, Noah A. Rosenberg, Ripan S. Malhi

Corresponding authors: Noah A. Rosenberg, Ripan S. Malhi
Email: noahr@stanford.edu, malhi@illinois.edu

This PDF file includes:

Supplementary Methods
Supplementary References
Figures S1 to S6
Tables S1 to S5

SUPPLEMENTARY METHODS

Samples

The study design considered three groups of individuals, where new samples were taken for DNA sequencing and merged together with data from existing individuals. The three sets of new data include 8 individuals from the *Síi Túupentak* archaeological site, 6 individuals from the *Rummey Ta Kuččuwiš Tiprectak* site, and 8 people from the present-day Muwekma Ohlone population. For the 8 modern individuals, participants provided genealogical information for parents and grandparents, indicating known ancestors both from the Muwekma Ohlone population and from other populations (primarily Mexican, European/European American). The ancient individuals were selected based mainly on endogenous DNA percentage in previous DNA analysis of the individuals [1].

DNA Extraction, library preparation, and sequencing

DNA extractions of samples taken from historic and ancient individuals from the *Síi Túupentak* and *Rummey Ta Kuččuwiš Tiprectak* sites were completed in an ancient DNA laboratory facility at the Carl R. Woese Institute for Genomic Biology at the University of Illinois. Surface contamination from the skeletal samples was removed by wiping each with Takara DNA-OFF^{Tm}, followed by submerging in 6% sodium hypochlorite (full strength Clorox bleach) for 6 minutes. The bleach was removed and the samples were then rinsed twice with DNA-free Molecular Biology Grade H_2O (no detectable DNase, RNase) and once with isopropanol to remove any remaining bleach. The samples were then placed in a UV cross linker until dry. Approximately 0.12-0.20 ml of skeletal powder was obtained from each sample using a Dremel tool at low speeds to minimize the production of heat. The powder was then incubated in 4 ml of demineralization/lysis buffer (0.5 M EDTA, 33.3 mg/ml Proteinase K, 10% N-lauryl sarcosine) for 12-24 hours at 37°C. The digested samples were then concentrated to approximately 100 µl using Amicon centrifugal filter units. Following concentration, the digests were run through UV-irradiated yeast RNA pre-treated silica columns using the Qiagen PCR Purification Kit and eluted in 60 µl volume of DNA extract [2].

Approximately 50 µl of DNA extract was used to create a genomic library with adapters that contained a unique dual index for each library (NEBNext Multiplex Oligos). Genomic libraries were created using the NEB Ultra II DNA Library Prep Kit. The DNA extract was not sheared as the DNA is expected to be fragmented due to taphonomic processes. A 1:20 dilution of adapters was used, as the DNA concentration in the extract is presumably low. Multiple Ampure Bead XP clean ups were completed in an attempt to remove adapter-dimers that may have developed. A PCR amplification of genomic libraries was prepared in the ancient DNA laboratory and then transported to thermocyclers in the modern DNA laboratory, across campus, in a sealed environment. The genomic libraries were amplified for 8-12 cycles and were then cleaned with a Qiagen MinElute Purification Kit. Library quantification was performed using a dsDNA HS assay on a Qubit fluorometer. The quality of the libraries was assessed on the Agilent 2100 Bioanalyzer using the High Sensitivity DNA kit and sequenced on an Illumina HiSeq 4000 and Novaseq 6000 using 100 bp reads.

Alignment and quality control: modern samples

DNA extractions from present-day community members were taken from saliva samples collected using Oragene DNA Saliva Kits and DNA extraction kits from DNA Genotek under University of Illinois IRB protocol #10538. Following DNA extraction and quantification, DNA extracts were sequenced by Novagene (Davis, CA) to approximately 25x coverage. Read data was aligned to GRCh37/hg19 human genome reference sequences.

Alignment and quality control: ancient samples

The alignment pipeline is shown in Figure S1. We sequenced 14 ancient samples, producing read data for each sample. For each sample, we first trimmed adapters with Cutadapt (1.18) [3], using parameters -m 25, -e 0.12, and --trim-n. Next, we aligned the trimmed reads to the GRCh37/hg19 human genome reference sequence with bwa (0.7.17) [4], using the aln and samse commands. We sorted the aligned reads by genomic position with Picard's (2.23.6) SortSam [5], applying parameters SORT_ORDER=coordinate and COMPRESSION_LEVEL=0, and added a read group label to all reads arising from a single sample with AddOrReplaceReadGroups. We removed reads with mapping quality less than 25, including reads which did not align to the reference sequence, with samtools' (1.8) [6] command view and flags -F 4 -q 25. We then removed PCR duplicate reads with samtools' (1.8) rmdup. We assessed genome-wide coverage levels using mosdepth (0.2.7) [7] and tabulated alignment metrics with samtools (1.8) stats (Table S2).

We discarded samples 7b and 10 from the *Rummey Ta Kuččuwiš Tiprectak* site for having few or no reads after alignment. We found that the older *Rummey Ta Kuččuwiš Tiprectak* samples tended to have shorter read lengths than the more recent samples, from the *Síi Túupentak* site. This pattern is consistent with the older samples having accrued more damage, resulting in shorter reads. We used the proportion of reads aligned to the X and Y chromosomes to determine sex: samples with fewer than 4% of reads aligned to chromosome X and greater than 0.1% of reads aligned to chromosome Y were assigned to be male. The 12 remaining samples consisted of 9 females and 3 males.

Next, we performed quality control on the aligned reads, as shown in Figure S2. Ancient sequence reads are often damaged, resulting in deamination patterns with C>T substitutions at the 5' end of the read and G>A substitutions at the 3' end. To authenticate that the sequence reads were ancient in origin, we assessed deamination patterns with MapDamage (2.0) [8] (Figure S3). We observed elevated G>A substitutions at the 3' end of the reads, suggesting that the reads originate from ancient samples; because our DNA libraries were amplified with a Phusion polymerase that interferes with the characteristic C>T substitution pattern for ancient samples, we observed few C>T substitutions at the 5' end.

We estimated contamination rates in the ancient sequence data using two methods. In males, contamination rates can be estimated using X-chromosomal polymorphic sites that are outside the pseudoautosomal region. To apply this approach, we first tabulated alleles at polymorphic sites on the X chromosome, excluding the pseudoautosomal region, using ANGSD (0.933) [9] with flags -r X, -doCounts 1, -iCounts 1, -minQ 20. Next, to obtain an initial point estimate of the contamination rates, we applied the ANGSD utility contamination with flags -p 2 -b 5000000 -c 154900000 -k 1 -m 0.05 -d 3 -e 20 -h HapMapCEU.gz. We refined the point estimates with contaminationX [10], with the parameter oneCns=1 (Table S2).

Next we estimated contamination rates in both males and females using polymorphic sites on the mitochondrial genome. First, we converted reads that had aligned to the mitochondrial genome back to a fastq file using samtools' (1.8) fastq. Next, we used these converted reads to create a consensus sequence with Multiple Iterative Assembler (MIA) (1.0) [11] in two steps: (1) we used the reads to create a maln file with command mia and parameter -k 14, and (2) we used the maln file to create the consensus sequence in fastq format with command ma. Then mafft (7.453) [12] created a multiple sequence alignment between the reference and consensus sequences. Next, we realigned the mitochondrial reads to the consensus sequence with bwa (0.7.17) using commands aln and samse. Finally, contamMix (1.0-10) [13] with flag --baseseq 25 used the realigned reads and the multiple sequence alignment to estimate contamination

rates in the ancient sequence data (Table S2). The estimated contamination levels are below 5% for each of the 12 samples, so we moved forward with all 12 samples.

At the conclusion of these steps, we then had data on 12 ancient samples, 8 from *Síi Túupentak* (6 females, 2 males) and 4 from *Rummey Ta Kuččuwiš Tiprectak* (3 females, 1 male). The mean number of aligned reads per sample was 88,374,857, with mean genomic coverage 2.35 (Table S2).

<u>Creating a dataset of published ancient and modern individuals</u>

We combined our new sequence data on 8 modern Muwekma Ohlone individuals and 12 ancient individuals with previously published data. We downloaded publicly available genetic data from 291 individuals, including 68 ancient and 223 modern individuals (Table S3). One of the 68 previously reported individuals was an individual from *Síi Túupentak*, from the same excavation as the *Síi Túupentak* individuals newly reported in this study; this individual, with sample label Ala1 (site Burial 1 median date 273 cal BP, and a two standard deviation range of 309-266 cal BP) was reported by [14]. In our analyses, this individual is grouped together with the newly reported individuals from *Síi Túupentak*.

The data that we merged with the new sequences came in three formats. For all ancient samples and some modern samples, we downloaded the sequencing read data (bam files). These samples were all whole-genome sequenced. Samples that came from the Human Genome Diversity Project (HGDP) and the Simons Genome Diversity Project (SGDP) were whole-genome sequenced to high coverage, resulting in large bam files, so we elected to download the variants as vcf files. The modern individuals from [15] were genotyped on an Illumina Human610-Quad array, and the genotypes were recorded in plink format (a .ped file).

Combining the 291 previously published samples with the additional 8 modern Muwekma Ohlone individuals and 12 ancient individuals resulted in 311 samples in total (Table S4).

<u>Data merging and genotype likelihood estimation</u>

The samples from [15] were genotyped on an Illumina Human610-Quad array and included 544,384 SNPs. We filtered out SNPs that are no longer mapped in dbSNP (v151); we also filtered out SNPs for which the strand assignment was ambiguous in the data of Verdu et al. (2014), resulting in 474,317 SNPs. At the remaining SNPs, we converted genotype calls to likelihoods in the following manner: the called genotype was assigned probability 1 and the other two genotypes were assigned probability 0. At sites with missing data, the three genotypes were assigned equal probability.

For the samples from the HGDP and SGDP, we extracted the phred scaled likelihoods of genotype calls from their vcf files at the 474,317 sites reported in the data of [15]. We converted the phred-scaled likelihoods to genotype likelihoods.

For the remaining samples in our dataset (present day Muwekma Ohlone; modern samples other than HGDP, SGDP, and [15]; all ancient samples), we had sequencing data in the form of bam files (Table S3). For these samples, we estimated genotype likelihoods with ANGSD (0.933) [9] and parameters -GL 2, -doMaf 1, -doGlf 2, -doMajorMinor 4, -minMapQ 25, -minQ 20, -checkBamHeaders 0 at the 474,317 sites. We merged the output with the converted likelihoods from the HGDP, SGDP, and [15] to create the full dataset of genotype likelihoods for all 311 individuals.

For detailed analysis of the context of our new samples, we also extracted a subset of 165 individuals, which on the basis of initial analysis of the full set of 311 individuals, possessed the greatest interest for understanding ancestry in the newly sampled ancient and modern individuals (Table S4). This subset excludes distant Arctic and Siberian regions and most South American individuals; it includes ancient individuals from Canada, the United States, Mexico, and the Lagoa Santa site in South America, and modern individuals from Europe, California, Mexico, and Colombia.

Principal components analysis (PCA)

We performed principal components analysis for both the full dataset of 311 individuals and the subset of 165 individuals, employing all 474,317 SNPs. We used pcangsd (0.985) [16] to estimate the covariance matrix of individual genotype vectors from the genotype likelihoods, using parameter -iter 500 to ensure convergence. The authors of pcangsd recommend setting the parameter e=k-1, where k is the number of clusters that best fit the data in a STRUCTURE-type analysis. We used -e 8 for the full dataset and -e 4 for the subset. From the covariance matrix estimated by pcangsd for the full data and subset, we used the eigen function in R (4.0.3) to calculate eigenvectors (principal components) and eigenvalues.

LD pruning

For the full dataset of 311 individuals at 474,317 SNPs, we estimated pairwise linkage disequilibrium. We used ngsLD (1.1.0) [17] to estimate pairwise LD from the genotype likelihoods using parameters --max_kb_dist 500 to only estimate LD between SNPs within 500 kb of each other, and parameter --min_maf 0.05 to only consider SNPs with minor allele frequency at least 5%. ngsLD estimates four measures of LD, and we chose r^2 as calculated by the EM algorithm. From the pairwise LD estimates, we next pruned our SNPs using the script prune_graph.pl included with ngsLD. To prune SNPs owing to pairwise $r^2<0.1$, we used parameters --min_weight 0.1, --max_kb_dist 500, and --weight_type a. After pruning, 85,659 SNPs remained.

Population clustering analysis

We used NGSadmix [18] to perform population clustering analysis on genotype likelihoods from the 85,659 SNPs that remained after LD pruning. For each tested number of clusters K, we performed the clustering 10 independent times, running NGSadmix with parameters -minMaf 0.05, -maxiter 10000, and -tol 0.000001. For the full dataset of 311 individuals, we considered K ranging from 4 to 12, with parameter -minInd 35. For the subset of 165 individuals, we considered K from 3 to 6, with parameter -minInd 15. In the full dataset, we found that values of K>10 produced new clusters consisting of just 2 or 3 individuals, and we disregarded these values in subsequent analysis. We also observed a similar phenomenon in the subset for values of K>5, and we disregarded those values as well.

For the full dataset and the subset, for each value of K, we evaluated the fit of K to the dataset using evalAdmix (0.9) [19]. Using the allele frequency estimates and q-matrix output from NGSadmix, evalAdmix calculates the correlation of the residual difference between the true genotypes and the genotypes predicted by NGSadmix. A lower correlation indicates a closer fit of the model to the data. In addition to visually inspecting correlation matrices produced by evalAdmix, we also compared the total absolute pairwise correlations, finding that the value with minimal correlation was K=10 in the full dataset and K=5 for the subset (Figure S4). These observations supported our choice to focus on K values less than or equal to 10 in the full dataset and less than or equal to 5 in the subset.

We next evaluated the clustering solutions inferred by NGSadmix across the 10 replicates for each value of *K* from 4 to 10 in the full dataset and *K* from 3 to 5 in the subset. We ran CLUMPP (1.1.2) [20] with parameters DATATYPE 0, M 2, W 0, S 2, and GREEDY_OPTION 2, and REPEATS 1000. Next, for each value of *K*, following [15], we clustered the runs based on pairwise G' values greater than 0.9. For the majority cluster of each *K* value, which contained the most runs, we reran CLUMPP with the same parameters to produce an averaged clustering solution for display in figures.

<u>Relatedness</u>
We checked for close relationships between our 12 newly sequenced ancient individuals and 8 modern individuals using NGSremix [21]. We chose NGSremix because the 8 present-day individuals possess admixture, which NGSremix accommodates in relatedness estimation. Relatedness coefficients were estimated from genotype likelihoods at 85,659 SNPs described above in "LD pruning" taking into account the output from NGSadmix at *K*=10, including the allele frequency and cluster membership estimates. We found no pairs more closely related than first cousins.

<u>Identity-by-state (IBS) segment calling</u>
We identified IBS segments between pairs of samples in four steps (Figure S5). First, we estimated genotype likelihoods in the ancient and modern samples. Second, we phased and imputed genotypes from the genotype likelihoods. Third, we called IBS segments from the phased genotypes. Fourth, in modern admixed individuals, we performed local ancestry assignment and identified IBS segments that lie on the Indigenous background, considering comparisons between modern samples and other modern samples, and between modern samples and ancient samples. This pipeline generated a list of IBS segments shared between ancient and modern individuals, restricting attention to the Indigenous-origin segments of the modern genomes.

Step 1. *Genotype likelihoods.* We began with all 80 ancient samples (68 previously published and 12 new) and a subset of 30 modern samples from California and Mexico, excluding the 22 individuals from the MXL population in the 1000 Genomes Project whose genotypes had previously been phased (Table S4). For this sample set, all samples have whole-genome sequences, and it is possible to use a much larger SNP set than in analyses that included larger numbers of samples. From the set of 2,504 individuals included in the phase 3 release of the 1000 Genomes Project, we identified 6,874,556 biallelic SNPs with a minor allele frequency (MAF) of at least 10% in the set of 2,504 individuals (1000 Genomes Project Consortium *et al.* 2015). Considering only those 6,874,556 biallelic SNPs that met this MAF threshold, in our set of 109 samples we estimated genotype likelihoods at those SNPs. For this estimation, we used ANGSD (0.933) [9] with flags -doBcf 1, -doGeno -4, -doPost 2, -doMajorMinor 3, -GL 1, -doMaf 1, -doCounts 1, -minMapQ 25, and -minQ 20, -trim 2. Note that the option -trim 2 trims two base pairs from each end of a read prior to imputation.

Step 2. Imputation and phasing. From the estimated genotype likelihoods at the 6,874,556 SNPs in the 109 individuals, we used GLIMPSE (1.1.0) [22] to phase and impute genotypes. First, using GLIMPSE, we divided the genome into overlapping regions with the chunk tool, using options --window-size 2000000, --window-count 2000, --buffer-size 200000, and --buffer-count 200. Next, we phased the overlapping regions, iteratively updating the genotype likelihoods; this step used the phase tool with parameter --main 15 and the 1000 Genomes phased genotypes from Step 1 as a reference. We then concatenated the phased overlapping regions using the ligate tool. Finally, we obtained phased, imputed genotypes for the samples; this step proceeded by sampling haplotypes from the ligated genomes using the sample tool

with option --solve. We considered three versions of the resulting data, examining all genotypes as well as genotypes filtered by the GLIMPSE INFO metric for imputation quality at threshold 0.5 (within the commonly used 0.3-0.5 range for the quality threshold) and a more stringent level of 0.9 (Figure S6).

Step 3. IBS calling. To call IBS segments, we merged the phased and imputed genotypes on 110 individuals with the phased genotypes from the 22 MXL individuals from the 1000 Genomes Project at the same SNPs. We called IBS segments in this combined data set using hap-IBD (1.0) [23], with parameters min-seed=1.0, max-gap=1000, min-extend=0.2, min-output=2.0, min-markers=100, min-mac=144; these settings are recommended by the authors of hap-IBD for whole-genome sequencing data.

Step 4. Restriction to Indigenous segments. For the modern individuals, we merged the phased and imputed genotypes from Step 2 with the phased genotypes of individuals from the MXL population from the 1000 Genomes Project, as used in Step 3, as well as the IBS individuals from 1000 Genomes Project. We estimated local ancestry in European and Indigenous sources in the modern samples with rfmix (2.03-r0) [24], using parameters -e 3, -n 5, and --reanalyze-reference, which allows reference samples to be somewhat admixed. For this step, 107 samples from the 1000 Genomes "Iberian populations in Spain" were used as the reference sample for Europeans and the 22 modern Mexican samples from Step 2 were used as the reference sample for Indigenous samples. This step assigned some segments of the genome as Indigenous in origin; we then restricted consideration of IBS segments identified in Step 3 to regions in which both haplotypes in a pair were assigned Indigenous ancestry. Thus, we identify IBD segments shared by modern individuals, considering those IBS segments that fall on an Indigenous background.

Finally, for each pair of individuals, we summed the length of IBS segments shared, and divided this length by $2p_1+2p_2$, where p_1 and p_2 are the proportions of the genome that are Indigenous in origin for individuals 1 and 2, respectively. This computation controls for differences in admixture proportions among the modern individuals. Because ancient individuals predate European contact (and show no signal of European ancestry in the analyses of recent admixture), they are assigned a proportion of 1. The three approaches to filtering (unfiltered, threshold 0.5, threshold 0.9) produce similar IBS sharing patterns; the analysis with unfiltered genotypes appears in Figure 6, and analyses with filtered genotypes appear in Figure S6.

f_4 statistic

To compare genetic affinity between various ancient and modern groups, we computed the f_4 statistic. We first called genotypes at the same set of 6,874,556 biallelic SNPs used in the identity-by-state analysis above. For the ancient individuals, at these SNPs, haploid genotypes were called from the aligned reads (bam files) using ANGSD (0.933) [9] with flags -doHaploCall 1, -doMajorMinor 3, -doCounts 1, -minMapQ 25, -minQ 20, -trim 3. For modern individuals, diploid genotypes were called with ANGSD using flags -doPlink 2, -doGeno -4, -doPost 2, -doMajorMinor 3, -GL 1, -doCounts 1, -doMaf 1, -minMapQ 25, -minQ 20. Ancient and modern genotypes were merged with the plink [25] command --bmerge and option --merge-equal-pos. The f_4 statistic and associated significance tests were computed between various population combinations with the function qpdstat in ADMIXTOOLS 2 [26]. Values appear in Table S5.

<u>SUPPLEMENTARY REFERENCES</u>

[1] T. Buonasera *et al.*, A comparison of proteomic, genomic, and osteological methods of archaeological sex estimation. *Sci. Rep.* **10**, 11897 (2020).

[2] A. E. Doran, D. R. Foran, Assessment and mitigation of DNA loss utilizing centrifugal filtration devices. *Forensic Sci. Int. Genet.* **13**, 187-190 (2014).

[3] M. Martin, Cutadapt removes adapter sequences from high-throughput sequencing reads. *EMBnet.journal* **17**, 10-12 (2011).

[4] H. Li, R. Durbin, Fast and accurate short read alignment with Burrows-Wheeler transform. *Bioinformatics* **25**, 1754-1760 (2009).

[5] *Picard toolkit*, Broad Institute (2019).

[6] H. Li *et al.*, The sequence alignment/map format and SAMtools. *Bioinformatics* **25**, 2078-2079 (2009).

[7] B. S. Pedersen, A. R. Quinlan, Mosdepth: quick coverage calculation for genomes and exomes. *Bioinformatics* **34**, 867-868 (2018).

[8] H. Jónsson, A. Ginolhac, M. Schubert, P. L. F. Johnson, L. Orlando, mapDamage2.0: fast approximate Bayesian estimates of ancient DNA damage parameters. *Bioinformatics* **29**, 1682-1684 (2013).

[9] T. S. Korneliussen, A. Albrechtsen, R. Nielsen, ANGSD: analysis of next generation sequencing data. *BMC Bioinformatics* **15**, 356 (2014).

[10] J. V. Moreno-Mayar *et al.*, A likelihood method for estimating present-day human contamination in ancient male samples using low-depth X-chromosome data. *Bioinformatics* **36**, 828-841 (2020).

[11] R. E. Green *et al.*, A complete Neandertal mitochondrial genome sequence determined by high-throughput sequencing. *Cell* **134**, 416-426 (2008).

[12] K. Katoh, D. M. Standley, MAFFT multiple sequence alignment software version 7: improvements in performance and usability. *Mol. Biol. Evol.* **30**, 772-780 (2013).

[13] Q. Fu *et al.*, A revised timescale for human evolution based on ancient mitochondrial genomes. *Curr. Biol.* **23**, 553-559 (2013).

[14] C. L. Scheib *et al.*, Ancient human parallel lineages within North America contributed to a coastal expansion. *Science* **360**, 1024-1027 (2018).

[15] P. Verdu *et al.*, Patterns of admixture and population structure in native populations of Northwest North America. *PLoS Genet.* **10**, e1004530 (2014).

[16] J. Meisner, A. Albrechtsen, Inferring population structure and admixture proportions in low-depth NGS data. *Genetics* **210**, 719-731 (2018).

[17] E. A. Fox, A. E. Wright, M. Fumagalli, F. G. Vieira, ngsLD: evaluating linkage disequilibrium using genotype likelihoods. *Bioinformatics* **35**, 3855-3856 (2019).

[18] L. Skotte, T. S. Korneliussen, A. Albrechtsen, Estimating individual admixture proportions from next generation sequencing data. *Genetics* **195**, 693-702 (2013).

[19] G. Garcia-Erill, A. Albrechtsen, Evaluation of model fit of inferred admixture proportions. *Mol. Ecol. Resour.* **20**, 936-949 (2020).

[20] M. Jakobsson, N. A. Rosenberg, CLUMPP: a cluster matching and permutation program for dealing with label switching and multimodality in analysis of population structure. *Bioinformatics* **23**, 1801-1806 (2007).

[21] A. K. Nøhr *et al.*, NGSremix: A software tool for estimating pairwise relatedness between admixed individuals from next-generation sequencing data. *G3 Genes Genomes Genet.* **11**, jkab174 (2021).

[22] S. Rubinacci, D. M. Ribeiro, R. J. Hofmeister, O. Delaneau, Efficient phasing and imputation of low-coverage sequencing data using large reference panels. *Nat. Genet.* 53, 120-126 (2021).

[23] Y. Zhou, S. R. Browning, B. L. Browning, A fast and simple method for detecting identity-by-descent segments in large-scale data. *Am. J. Hum. Genet.* **106**, 426-437 (2020).

[24] B. K. Maples, S. Gravel, E. E. Kenny, C. D. Bustamante, RFMix: a discriminative modeling approach for rapid and robust local-ancestry inference. *Am. J. Hum. Genet.* **93**, 278-288 (2013).

[25] S. Purcell *et al.*, PLINK: a tool set for whole-genome association and population-based linkage analyses. *Am. J. Hum. Genet.* **81**, 559-575 (2007).

[26] N. Patterson *et al.*, Ancient admixture in human history. *Genetics* **192**, 1065-1093 (2012).

[27] A. Leventhal, D. DiGiuseppe, D. Grant, R. Cambra, M. V. Arellano, S. Guzman-Schmidt, G. E. Gomez, A. Sanchez, Report on the analysis and temporal placement of an ancestral Muwekma Ohlone burial recovered from *'Ayttakiš 'Éete Hiramwiš Trépam-tak* [Place of Woman Sleeping Under the Pipe Site], CA-ALA-677/H located in the town of Sunol, Alameda County, California. Report prepared for the San Francisco Public Utilities Commission (2017).

[28] M. Rasmussen *et al.*, The genome of a Late Pleistocene human from a Clovis burial site in western Montana. *Nature* **506**, 225-229 (2014).

[29] 1000 Genomes Project Consortium, A global reference for human genetic variation. *Nature* **526**, 68-74 (2015).

[30] M. Raghavan *et al.*, Genomic evidence for the Pleistocene and recent population history of Native Americans. *Science* **349**, aab3884 (2015).

[31] M. Rasmussen *et al.*, The ancestry and affiliations of Kennewick Man. *Nature* **523**, 455-458 (2015).

[32] S. Mallick *et al.*, The Simons Genome Diversity Project: 300 genomes from 142 diverse populations. *Nature* 538, 201-206 (2016).

[33] J. Lindo *et al.*, Ancient individuals from the North American Northwest Coast reveal 10,000 years of regional genetic continuity. *Proc. Natl. Acad. Sci. U. S. A.* **114**, 4093-4098 (2017).

[34] J. V. Moreno-Mayar *et al.*, Terminal Pleistocene Alaskan genome reveals first founding population of Native Americans. *Nature* **553**, 203-207 (2018).

[35] J. V. Moreno-Mayar *et al.*, Early human dispersals within the Americas. *Science* **362**, aav2621 (2018).

[36] P. Flegontov *et al.*, Palaeo-Eskimo genetic ancestry and the peopling of Chukotka and North America. *Nature* **570**, 236-240 (2019).

[37] A. Bergstrom *et al.*, Insights into human genetic variation and population history from 929 diverse genomes. *Science* **367**, eaay5012 (2020).

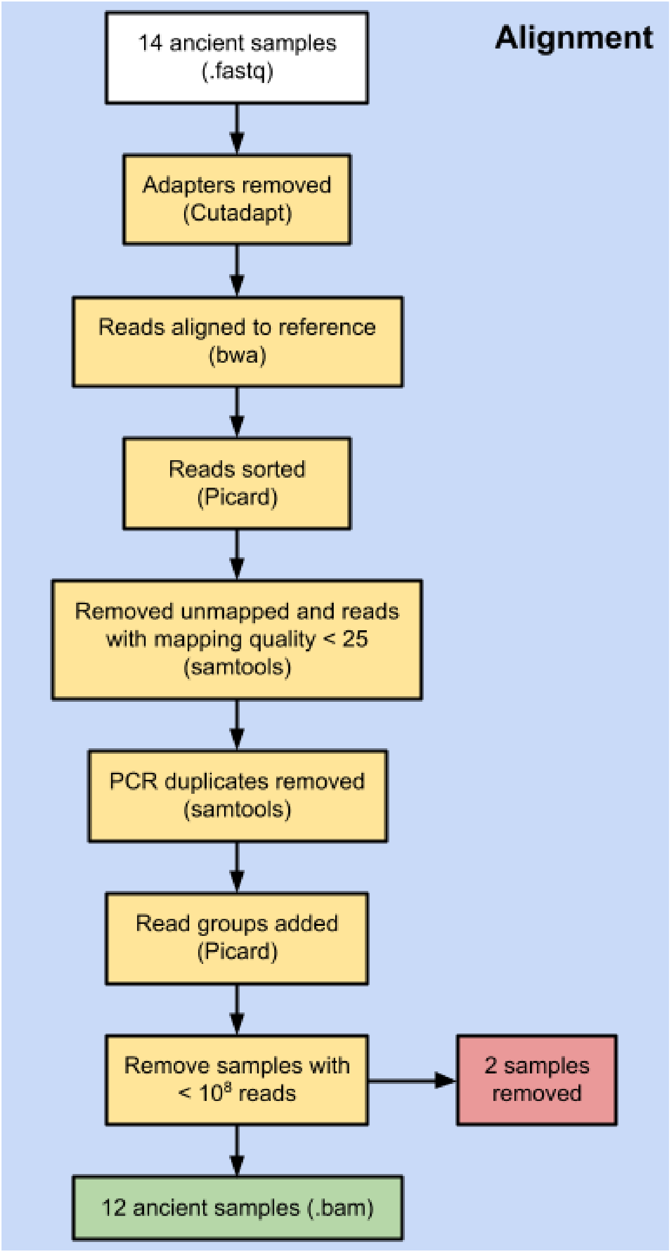

Figure S1: The read alignment pipeline for the newly sequenced ancient individuals in the study.

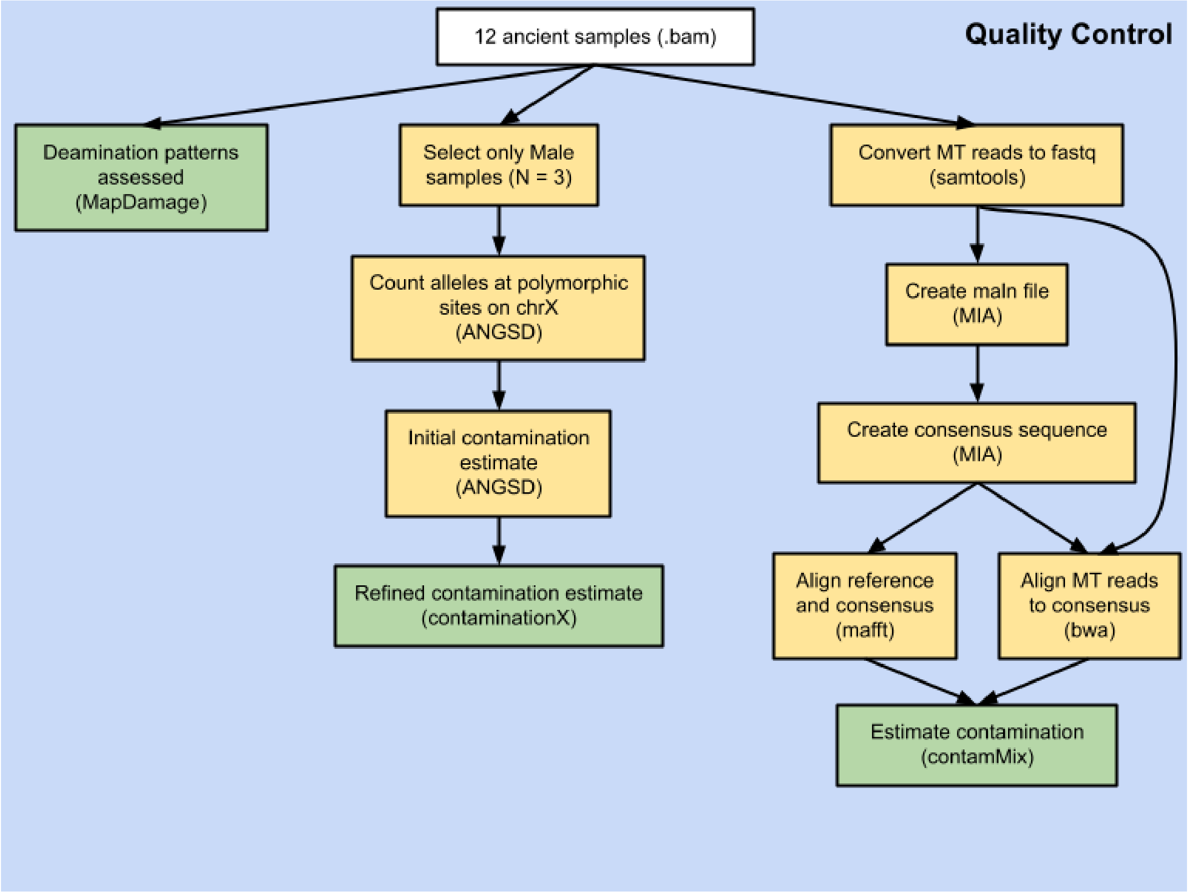

Figure S2: The quality control pipeline for the 12 newly sequenced individuals in the study.

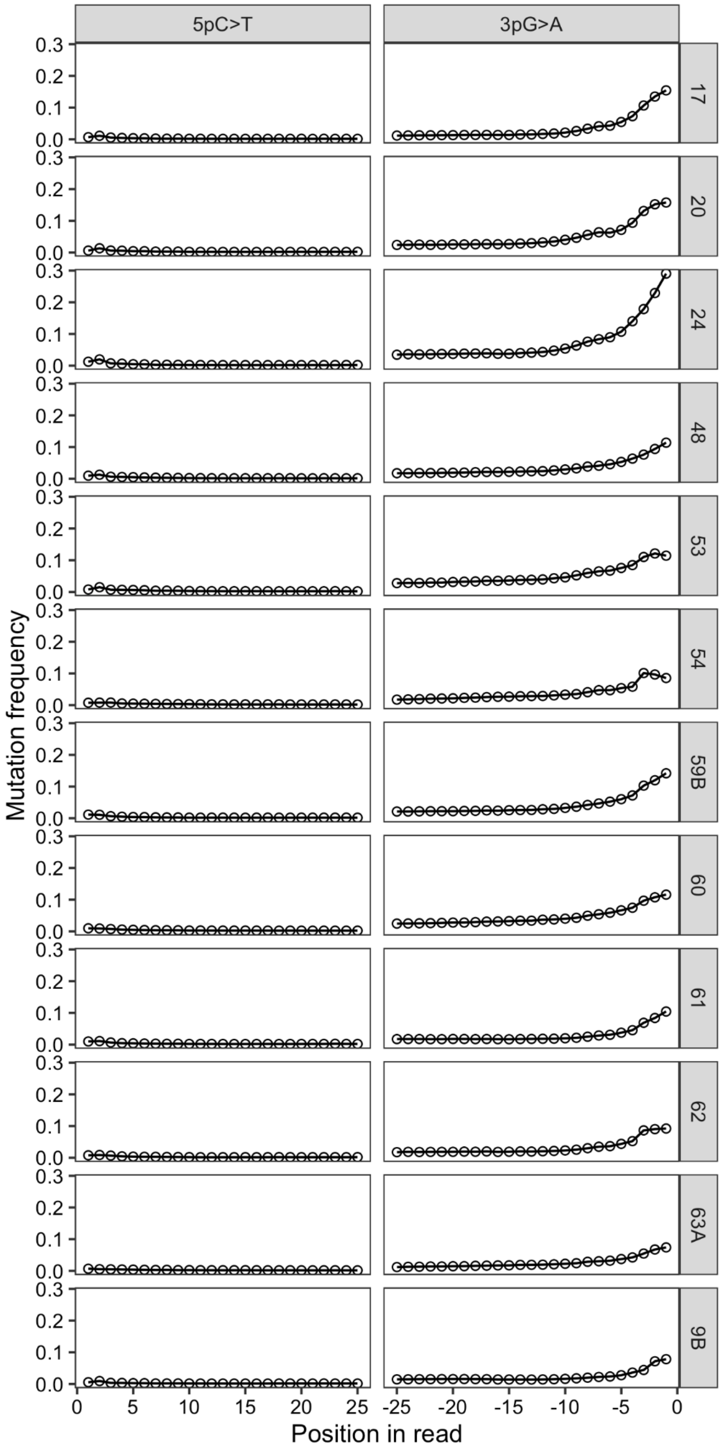

Figure S3: The damage patterns present in the 12 newly sequenced ancient samples, as estimated by MapDamage. The first column, 5pC>T, shows the rate of damage on the 5' end of the reads, observed as C>T mutations. Similarly, the second column, 3pG>A, shows the rate of damage on the 3' end of the reads, observed as G>A mutations. Sample numbers appear on the right.

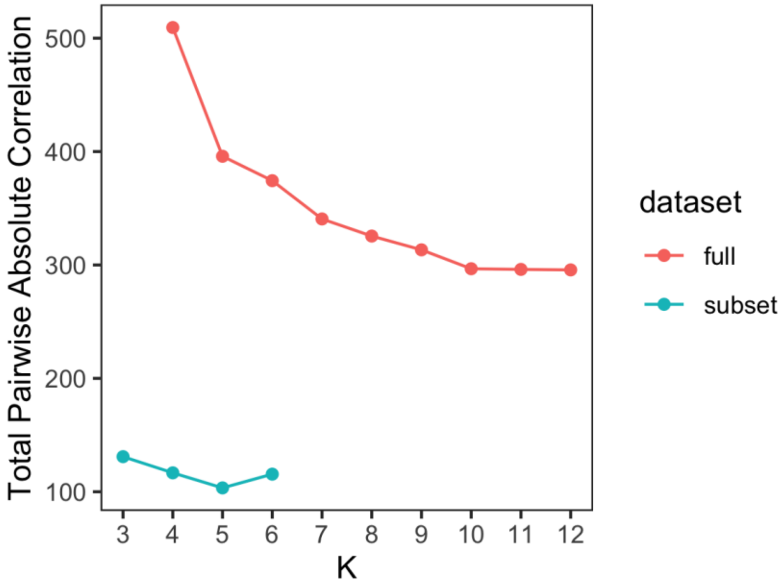

Figure S4: The total pairwise absolute correlation amongst individuals in the full dataset (*N*=311) and the subset (*N*=165), as calculated by evalAdmix [19]. For each value of *K*, evalAdmix compares the estimated genotype to the observed and calculates the residual, and then calculates the correlation of the residuals between pairs of individuals in the dataset. The value shown represents a sum across all pairs of individuals of this correlation at each value of *K* for the full dataset and the subset.

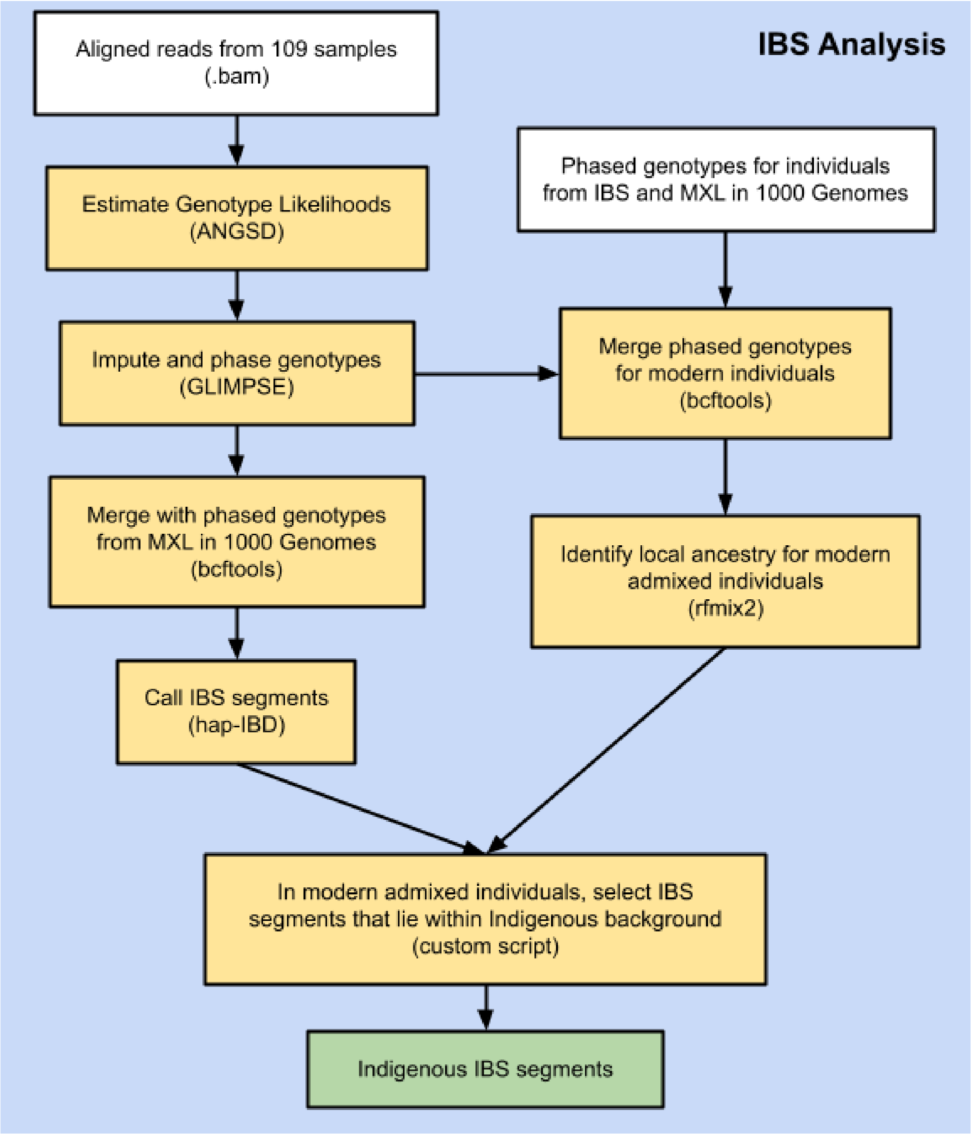

Figure S5: The procedure used to identify IBS segments for pairs of individuals.

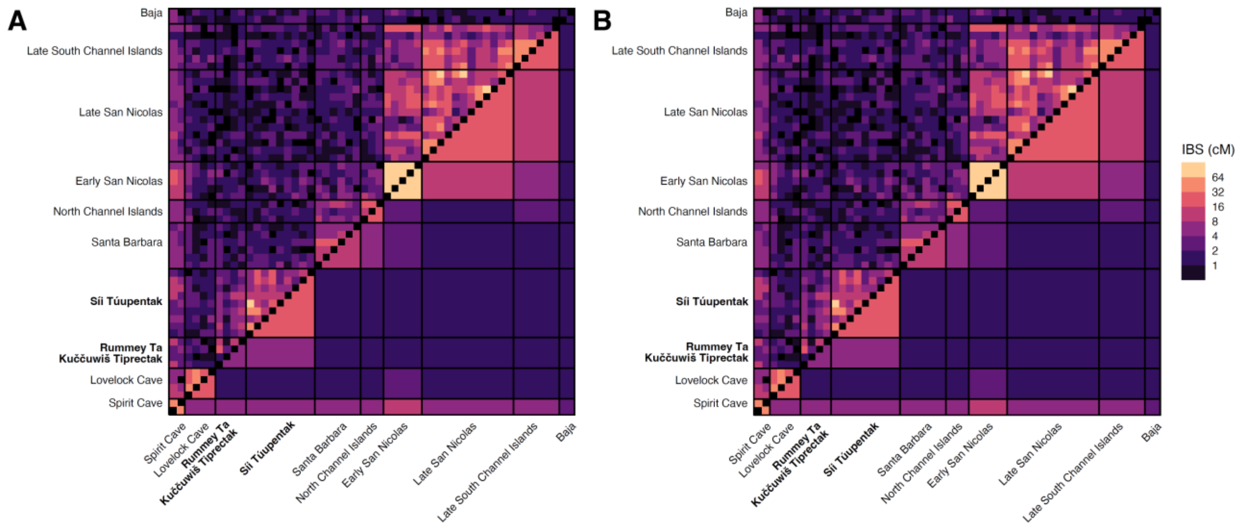

Figure S6: Total pairwise identity-by-state (IBS) segment sharing for 53 ancient individuals from Nevada, California, and the Baja peninsula, on the basis of imputed genotypes selected according to quality filters. (A) Quality threshold 0.5. (B) Quality threshold 0.9. Figure design follows Figure 6. The upper triangle of the matrix shows the total length of segments shared for pairs of individuals. The lower triangle shows mean pairwise values. In the triangularly shaped regions incident to the diagonal, the mean is taken across pairs within a population; rectangles off the diagonal show means across pairs, one from one population and one from another population.

Table S1: Sunol Area Ohlone Historical Events Timeline, with emphasis on the Native People -- the Muwekma Ohlone -- of Mission San Jose. Key sources drawn from: (1) Arellano, Monica V., Alan Leventhal, Charlene Nijmeh, Shelia Guzman Schmidt and Gloria Arellano Gomez. 2020. “Ethnohistory, Historic Ties, and Tribal Stewardship of Sunol/Pleasanton, Santa Clara Valley, and Adjacent Areas.” In Byrd, B.F. et al., Protohistoric Village Organization and Territorial Maintenance: The Archaeology of Síi Túupentak (CA-ALA-565/H) in the San Francisco Bay Area, pp. 17-56. Center for Archaeological Research, Davis, California. (2) Milliken, Randall. 2002. California Indians of the Mission San Jose Outreach Area. Far Western Research Group, Davis, CA. Prepared for Archaeo Archaeological Consultants, Fremont, California.

**Event**	**Period**	**Common Era (CE)**	**Notes**
Rummey Ta Kuččuwiš Tiprectak (CA-ALA-704/H, “Place of the Stream of the Lagoon Site“) Occupied	Pre-Contact	490 BCE – 1775 CE	Most evidence from 490 BCE-340 CE; near confluence of 2 major rivers along a major E-W travel route
**’Ayttakiš ’Éete Hiramwiš Trépam-tak** (CA-ALA-677/H, "Place of Woman Sleeping Under the Pipe Site") Occupied	Precontact	1263-1435	Additional settlement in close vicinity of two sites in this study
Síi Túupentak (CA-ALA-565/H, “Place of the Water Round House Site”) Occupied	Pre-Contact to Early Contact	1335-1805	Most evidence from 1410 onward, settled at confluence of 2 major rivers near mouth of Niles Canyon, near ***Rummey Ta Kuččuwiš Tiprectak***
Drake lands in Marin	Early Contact	1579	35-day stay by English, reported that 13+ left behind & most walked back to Baja
Cermeno lands in Marin	Early Contact	1595	21-day stay by Spanish
Portola Expedition in San Francisco Bay	Spanish	1769	Went into southeast Bay Area
Monterey Presidio and Mission San Carlos Borromeo founded	Spanish	1770	Headquarters of Alta California Governor
Fages-Crespi Expedition passes through Sunol	Spanish	1772	Saw "good village" in Sunol area, Thursday April 2 - probably ***Síi Túupentak;*** named area Santa Coleta and recommended a mission be established there
Mission San Francisco Asis (MSF) and Presidio (fort) founded	Spanish	1776	
Mission Santa Clara (MSC) founded	Spanish	1777	
Mission San Jose (MSJ) founded	Spanish	1797	6 km southwest of ***Síi Túupentak***
First two Causen Ohlone inhabitants go to Mission Santa Clara	Spanish	1795	Ohlone name of tribal territorial-community area centered on Sunol
200 Causen (including ***Síi Túupentak*** villagers) go to Mission San Jose	Spanish	1795-1807	No other Causen go in missions after that
3500 Yokuts, Miwok and Patwin come to Mission San Jose	Spanish-Mexican	1809-1834	Mission indigenous population 80% Ohlone in 1809; 4% Ohlone in 1835 (comingling of groups and traditions)
Mission San Jose has largest Native population of all missions	Spanish	1820	Indigenous population reaches 1700 (MSF only 650); MSJ remains largest after that
Mexico takes over	Mexican	1822	Mission power declines, in 1824 mission lands opened to new colonizers
Síi Túupentak re-occupied	Mexican	1831–1838	Southeast edge of site
Secularization Act closing missions takes effect	Mexican	1836	Mission lands, infrastructure, and animals to private individuals; law passed in 1834 overruled Spanish law that had planned to give land to back to indigenous population; MSJ closed as landholding entity, now under civil control (remained as church and recording life events)
Mission San Jose population disperses	Mexican	1837-1839	Mission population drops from 1550 to 585 (most of the indigenous inhabitants go east to Central Valley/foothills; even those that stay had to feed themselves)
Pico and Bernal get land grant that includes Sunol	Mexican	1839	Rancho el Valle de San Jose, 48,436-acres
Suñol Adobe established	Mexican	early 1840s	Ranch "at intersection of three roads", on top of Ohlone site ***Rummey Ta Kuččuwiš Tiprectak*** (impacting site deposits), and close to ***Síi Túupentak***
Classic Rancho Period	Mexican	1840s	Indigenous population were the primary labor pool, worked as servants, laborers, cowboys at ranches (often at pre-contact villages locations such as Sunol), referred to as serfs/slaves in historical documents; Missions records document indigenous individuals at ranchos, including 2 from Sunol ranch
Indigenous community established north of MSJ in San Leandro area	Mexican	1844	led by Ohlone Buenaventura; land license granted by MSJ priest

US takes over, California becomes a state	American	1850	January 1851: Governor Burnett says "a war of extermination will continue to be waged between the races until the Indian race should become extinct"; subsequently bounty ads for dead Indians placed in newspapers; law allowed Indian children to be kidnapped for use as slaves
Hadsell acquires Sunol area ranch lands and resides there	American	1851	
US treaties (which were never ratified) signed by many California tribes	American	1851-1852	Tribes near San Francisco Bay missions largely ignored as not viewed a threat to settlers/miners (yet tribe states "Muwekma Ohlone Tribe were to be the intended beneficiaries of two of the treaties: E. Treaty of Dent's and Ventine's Crossing, May 28, 1851 and M. Treaty of Camp Fremont, March 19, 1851.")
Use of Indigenous labor on ranches greatly declines	American	1850s-1860s	Newer American ranch owners tended to employ white immigrants (failed gold rushers), and indigenous workers driven off ranches (only some Mexican owners continued to do so, such as Bernal in Pleasanton area)
Traditional Indigenous practices continued	American	1850s-1860s	Dances houses and sweat lodges persisted, such as in San Leandro and Alisal area (near Sunol)
Pleasanton and Niles Canyon area refuges for East Bay Indigenous Community	American	1860s-1910s	Largest group of former MSJ & MSC Indigenous inhabitants resided (from 1840s onward) at an ancestral village on Bernal Ranch at Alisal (the Alders) southwest of Pleasanton, <5 km north of ***Síi Túupentak;*** 100 indigenous descendent families of MSJ living in greater bay area in late 1800s
Final segment of the First Transcontinental Railroad completed through Sunol and Niles Canyon	American	1870	Work started in 1866; provided first rail connection between the San Francisco Bay area and the rest of the United States
Indigenous ceremonial revitalization	American	1870s	Dance ceremonies thrive/resurge especially at Alisal, but also with indigenous community members living on San Leandro, Niles, and Livermore rancherias; local version of Ghost Dance merged with local Kukśu religious practices, thrived and exported from Alisal
Spring Valley Water Company acquires Sunol ranch land of Haskell	American	1875	Muwekma Ohlone worked for company
Hearst acquires land that includes Alisal and Niles canyon indigenous settlements	American	1886	US Senator George Hearst and Phoebe Apperson Hearst purchased a large parcel that included Alisal Rancheria; allowed an indigenous community of approximately 125 to continue living and working for them
Indigenous community living on Hearst Land near Verona rail stop	American	1886-1910+	Western Pacific Railroad constructed a rail line to serve the Hearst estate in 1910 and named the station Verona (after the Hearst mansion Hacienda del Pozo de Verona); <3 km north of ***Síi Túupentak***
Sunol Water Temple and water filters built	American	1904	removes a portion of ***Síi Túupentak***
Federal reconsideration of unratified 1851-52 treaties to provide land to homeless CA tribes	American	1905-1937	Includes Verona Band of Alisal, Livermore and Niles Canyon, recognized descendants of MSJ
Muwekma children born on the Alisal and Sunol Rancherias	American	1900-1917	Marine family lineage children baptized at Mission San Jose and St. Augustine's Pleasanton
Alisal and possibly Niles Canyon indigenous settlements abandoned	American	1914-1916	at Alisal, last chief Jose Antonio died in 1897 (recorded at MSJ, buried at indigenous cemetery there); last Kukśu religious dance in 1897, dance house torn down in 1900
Muwekma Men enlist in the US Armed Forces: Army, Navy & Marines	American	1914-1920	All six men served overseas
California indigenous population legally became US citizens	American	1924	
Muwekma Enrollment with the Bureau of Indian Affairs	American	1928–1932	California Indian Jurisdictional Act of 1928
Muwekma Children in Indian Boarding Schools	American	1931–1946	Sherman Institute, Riverside, CA and Chemawa, Salem, OR
Verona Band removed from consideration for homesite purchase	American	1927	Sacramento Superintendent, Dorrington, removes 135 California Indian Bands
Muwekma men serve overseas Army, Navy, Marines, and Army Air	American	1940-1957	101st and 82nd Airborne, Patton's 3rd Army Tank Div, 41st Inf Div, 640th Tank Dest.
San Francisco buys Water Company lands	American	1930	SFPUC administered these as agricultural lands
Muwekma Ohlone enroll in BIA Second Enrollment Act	American	1948–1957	Settlement $150.00 per enrolled head of household for 8.5 million acres of land
Muwekma Ohlone enroll in BIA Third Enrollment Act	American	1968-1971	Settlement $668.61 per enrolled head of household for 64 million acres of land

Muwekma organize to petition US to regain Federal Recognition	American	1980-2013	Muwekma obtains formal determination of previous recognition from BIA
SFPUC Formalizes Plans to build Watershed Center on ***Síi Túupenta***	American	2015	SFPUC working in collaborative partnership with Muwekma leadership
California Governor Gavin Newsom acknowledges and apologized for state-sponsored genocide	American	2019	In June the Governor stated: "That’s what it was, a genocide. No other way to describe it. And that’s the way it needs to be described in the history books."

Table S2: Archaeological site, sample age, information, alignment metrics, coverage, and contamination estimates for the 14 ancient individuals sequenced in this study. All dates were calibrated with a mixed marine curve based on established protocols using individual δ13C values. Samples 10 (no reads) and 7b (few reads) were not used in the genetic data analysis. Sample 20 had few reads and could only be assigned to mtDNA haplogroup M (which includes haplogroup C and D). The mtDNA haplogroups for the present-day individuals are B2x, B2, H7, B2, B2, A2c, D1, U5b1c1 for individuals M01-M08 (in order). The chrY haplogroups for the present-day male individuals are Q1a2a1b, R1b1a2a2, O2a2b1a1a2 for individuals M01-M03 (in order); individuals M04-M08 are female. We note that upon completion of the analysis in this study, another burial site, ’Ayttakiš ’Éete Hiramwiš Trépam-tak [Place of Woman Sleeping Under the Pipe Site] CA-ALA-677/H was being excavated [27]. DNA analysis of ancestral remains from this site are planned for a future study.

Sample	Site	Mixed Marine Median Probability*	Mixed Marine 2δ Range	Sex	Age	Reads mapped	Mean read length	Mean coverage	contaminationX	contamMix	MT haplogroup	chrY haplogroup
7b	*Rummey Ta Kuččuwiš Tiprectak*	1832	1890-1730	F	20-25	4409	68					
9b	*Rummey Ta Kuččuwiš Tiprectak*	1867	1946-1806	F	30-40	63837176	87	1.84	NA	0.04%	C1f	NA
10	*Rummey Ta Kuččuwiš Tiprectak*	289	318-267	F	27-33	0						
17	*Rummey Ta Kuččuwiš Tiprectak*	1826	1883-1728	M	<1	21118373	54	0.37	0.66%	0.81%	D1	Q1a2a1a1
20	*Rummey Ta Kuččuwiš Tiprectak*	1905	1953-1858	F	1.5-2.5	2434870	74	0.06	NA	2.50%	M	NA
24	*Rummey Ta Kuččuwiš Tiprectak*	1889	1946-1824	F	40-45	207035286	63	4.31	NA	1.60%	B2b	NA
48	*Síi Túupentak*	601	652-548	F	25-34	266564813	87	7.66	NA	0.02%	D1	NA
53	*Síi Túupentak*	499	522-462	F	>60	120545025	85	3.4	NA	0.02%	D1	NA
54	*Síi Túupentak*	384	460-309	F	<1	105210222	84	2.94	NA	0.78%	D1	NA
59b	*Síi Túupentak*	383	438-347	F	>20	59201100	83	1.63	NA	3.96%	C1c1b	NA
60	*Síi Túupentak*	184	229-135	F	7-8	115612803	85	3.26	NA	0.18%	D1	NA
61	*Síi Túupentak*	511	529-488	M	40-44	15155789	85	0.42	0.26%	2.09%	C1c1b	Q1a2a1a1
62	*Síi Túupentak*	304	330-280	F	fetus	28881585	85	0.82	NA	0.65%	D1	NA
63a	*Síi Túupentak*	184	232-133	M	1-2	54901243	86	1.54	3.73%	0.18%	D1	Q1a2a1a1

Table S3: Summary of previously published individuals used in this study (N=291; 223 modern, 68 ancient). The samples with .bam files contained 130, the samples with genotypes in .vcf files contained 82, and the samples with genotypes in plink format contained 79.

**Strategy**	**Data format**	**Ancient / Modern**	**Region**	**Sample Size**	**Source**
WGS	sequences (.bam)	ancient	Alaska / Canadian Arctic	7	Flegontov *et al.* (2019)
WGS	sequences (.bam)	ancient	Pacific Northwest	4	Lindo *et al.* (2017)
WGS	sequences (.bam)	ancient	Alaska	2	Moreno-Mayar *et al.* (2018) A
WGS	sequences (.bam)	ancient	Pacific Northwest	1	Moreno-Mayar *et al.* (2018) B
WGS	sequences (.bam)	ancient	Nevada	6	Moreno-Mayar *et al.* (2018) B
WGS	sequences (.bam)	ancient	South America	5	Moreno-Mayar *et al.* (2018) B
WGS	sequences (.bam)	ancient	Greenland	1	Raghavan *et al.* (2015)
WGS	sequences (.bam)	ancient	East Canada	1	Raghavan *et al.* (2015)
WGS	sequences (.bam)	ancient	Mexico	1	Raghavan *et al.* (2015)
WGS	sequences (.bam)	ancient	North American	1	Rasmussen *et al.* (2015)
WGS	sequences (.bam)	ancient	North American	1	Rasmussen *et al.* (2014)
WGS	sequences (.bam)	ancient	Alaska	1	Scheib *et al.* (2018)
WGS	sequences (.bam)	ancient	East Canada	3	Scheib *et al.* (2018)
WGS	sequences (.bam)	ancient	San Francisco Bay Area	1	Scheib *et al.* (2018)
WGS	sequences (.bam)	ancient	Southern California	32	Scheib *et al.* (2018)
WGS	sequences (.bam)	ancient	Mexico	1	Scheib *et al.* (2018)
WGS	sequences (.bam)	modern	Siberia	10	Raghavan *et al.* (2015)
WGS	sequences (.bam)	modern	Alaska	3	Raghavan *et al.* (2015)
WGS	sequences (.bam)	modern	Greenland	2	Raghavan *et al.* (2015)
WGS	sequences (.bam)	modern	Mexico	22	The 1000 Genomes Project Consortium *et al.* (2015)
WGS	sequences (.bam)	modern	European	25	The 1000 Genomes Project Consortium *et al.* (2015)
WGS	genotypes (.vcf)	modern	Mongolian	9	Bergstrom *et al.* (2020)
WGS	genotypes (.vcf)	modern	Mexico	33	Bergstrom *et al.* (2020)
WGS	genotypes (.vcf)	modern	South America	23	Bergstrom *et al.* (2020)
WGS	genotypes (.vcf)	modern	Siberia	7	Mallick *et al.* (2016)
WGS	genotypes (.vcf)	modern	Mexico	6	Mallick *et al.* (2016)
WGS	genotypes (.vcf)	modern	South America	4	Mallick *et al.* (2016)
Array	genotypes (plink)	modern	Pacific Northwest	79	Verdu *et al.* (2014)

Table S4: The complete list of samples used in this study. The table contains 311 individuals, 165 of whom appear in the subset analysis. From the subset analysis, 132 also appear in the IBS analysis. Note that “San Nicolas” refers to San Nicolas Island, whereas “Santa Barbara” refers to the mainland near the city of Santa Barbara, rather than to Santa Barbara Island.

**Sample ID**	**Ancient / Modern**	**Label / Population**	**Region**	**Included in subset**	**Included in IBD analysis**	**Latitude**	**Longitude**	**Source**
I5319	ancient	Athabaskan	Alaska	F	T	62.95	-155.59	Flegontov et al. [36]
I5321	ancient	Athabaskan	Alaska	F	T	62.95	-155.59	Flegontov et al. [36]
I1118	ancient	Neo-Aleutian	Alaska	F	T	52.99	-169.71	Flegontov et al. [36]
I1125	ancient	Neo-Aleutian	Alaska	F	T	53.37	-167.83	Flegontov et al. [36]
I1127	ancient	Neo-Aleutian	Alaska	F	T	52.99	-169.71	Flegontov et al. [36]
I1129	ancient	Neo-Aleutian	Alaska	F	T	52.99	-169.71	Flegontov et al. [36]
523a-C	ancient	Palm Site	Alaska	F	T	60.5496	-151.299	Scheib et al. [14]
TrailCreek	ancient	Trail Creek	Alaska	F	T	65.79	-163.41	Moreno-Mayar et al. [35]
USR1	ancient	Upward Sun River	Alaska	F	T	64.22	-145.71	Moreno-Mayar et al. [34]
I10427	ancient	Canadian Arctic	Canada	F	T	69.4	-106.26	Flegontov et al. [36]
CK-13	ancient	Southwest Ontario	East Canada	T	T	42.233	-83.101	Scheib et al. [14]
LU-01	ancient	Southwest Ontario	East Canada	T	T	42.233	-83.101	Scheib et al. [14]
MARC1492	ancient	Southwest Ontario	East Canada	T	T	50	-66.7257	Raghavan et al. [30]
RM-83	ancient	Southwest Ontario	East Canada	T	T	43.86	-79.56	Scheib et al. [14]
Saqqaq	ancient	Saqqaq	Greenland	F	T	68.591	-51.288	Raghavan et al. [30]
B-3	ancient	Baja	Mexico	T	T	26.072	-111.84	Scheib et al. [14]
BC25	ancient	Baja	Mexico	T	T	23.428	-109.883	Raghavan et al. [30]
Anzick-1	ancient	Clovis	Montana	T	T	45.96	-110.66	Rasmussen et al. [28]
Lovelock1	ancient	Lovelock Cave	Nevada	T	T	39.96	-118.56	Moreno-Mayar et al. [35]
Lovelock2	ancient	Lovelock Cave	Nevada	T	T	39.96	-118.56	Moreno-Mayar et al. [35]
Lovelock3	ancient	Lovelock Cave	Nevada	T	T	39.96	-118.56	Moreno-Mayar et al. [35]
Lovelock4	ancient	Lovelock Cave	Nevada	T	T	39.96	-118.56	Moreno-Mayar et al. [35]
AHUR_2064	ancient	Spirit Cave	Nevada	T	T	39.24	-118.38	Moreno-Mayar et al. [35]
AHUR770c	ancient	Spirit Cave	Nevada	T	T	39.24	-118.38	Moreno-Mayar et al. [35]
19651	ancient	Big Bar	PNW	T	T	51.3	-121.77	Moreno-Mayar et al. [35]
302	ancient	Pacific NW Coast	PNW	T	T	54.3078	-130.355	Lindo et al. [33]
443	ancient	Pacific NW Coast	PNW	T	T	54.3078	-130.355	Lindo et al. [33]
939	ancient	Pacific NW Coast	PNW	T	T	54.3078	-130.355	Lindo et al. [33]
ShKa	ancient	Shuka Kaa	PNW	F	T	55.815	-132.823	Lindo et al. [33]
17	ancient	*Rummey Ta Kuččuwiš Tiprectak*	SF Bay Area	T	T	37.591	-121.884	*This study*
20	ancient	*Rummey Ta Kuččuwiš Tiprectak*	SF Bay Area	T	T	37.591	-121.884	*This study*
24	ancient	*Rummey Ta Kuččuwiš Tiprectak*	SF Bay Area	T	T	37.591	-121.884	*This study*
9b	ancient	*Rummey Ta Kuččuwiš Tiprectak*	SF Bay Area	T	T	37.591	-121.884	*This study*
48	ancient	*Síi Túupentak*	SF Bay Area	T	T	37.582	-121.87	*This study*
53	ancient	*Síi Túupentak*	SF Bay Area	T	T	37.582	-121.87	*This study*
54	ancient	*Síi Túupentak*	SF Bay Area	T	T	37.582	-121.87	*This study*
59	ancient	*Síi Túupentak*	SF Bay Area	T	T	37.582	-121.87	*This study*
60	ancient	*Síi Túupentak*	SF Bay Area	T	T	37.582	-121.87	*This study*
61	ancient	*Síi Túupentak*	SF Bay Area	T	T	37.582	-121.87	*This study*
62	ancient	*Síi Túupentak*	SF Bay Area	T	T	37.582	-121.87	*This study*
63	ancient	*Síi Túupentak*	SF Bay Area	T	T	37.582	-121.87	*This study*

Ala1-C	ancient	*Síi Túupentak*	SF Bay Area	T	T	37.582	-121.87	Scheib et al. [14]
Aconcagua	ancient	Aconcagua	South America	F	T	-32.654	-70.011	Moreno-Mayar et al. [35]
Sumidouro4	ancient	Lagoa Santa	South America	T	T	-19.54	-43.94	Moreno-Mayar et al. [35]
Sumidouro5	ancient	Lagoa Santa	South America	T	T	-19.54	-43.94	Moreno-Mayar et al. [35]
Sumidouro6	ancient	Lagoa Santa	South America	T	T	-19.54	-43.94	Moreno-Mayar et al. [35]
Sumidouro7	ancient	Lagoa Santa	South America	T	T	-19.54	-43.94	Moreno-Mayar et al. [35]
SN-17	ancient	Early San Nicolas	South California	T	T	33.2492	-119.5057	Scheib et al. [14]
SN-3	ancient	Early San Nicolas	South California	T	T	33.2492	-119.5057	Scheib et al. [14]
SN-32	ancient	Early San Nicolas	South California	T	T	33.2492	-119.5057	Scheib et al. [14]
SN-40	ancient	Early San Nicolas	South California	T	T	33.2492	-119.5057	Scheib et al. [14]
SN-44	ancient	Early San Nicolas	South California	T	T	33.2492	-119.5057	Scheib et al. [14]
SN-53	ancient	Early San Nicolas	South California	T	T	33.2492	-119.5057	Scheib et al. [14]
SN-54	ancient	Early San Nicolas	South California	T	T	33.2492	-119.5057	Scheib et al. [14]
SN-1	ancient	Late San Nicolas	South California	T	T	33.2492	-119.5057	Scheib et al. [14]
SN-10	ancient	Late San Nicolas	South California	T	T	33.2492	-119.5057	Scheib et al. [14]
SN-11	ancient	Late San Nicolas	South California	T	T	33.2492	-119.5057	Scheib et al. [14]
SN-13	ancient	Late San Nicolas	South California	T	T	33.2492	-119.5057	Scheib et al. [14]
SN-15	ancient	Late San Nicolas	South California	T	T	33.2492	-119.5057	Scheib et al. [14]
SN-16	ancient	Late San Nicolas	South California	T	T	33.2492	-119.5057	Scheib et al. [14]
SN-38	ancient	Late San Nicolas	South California	T	T	33.2492	-119.5057	Scheib et al. [14]
SN-50	ancient	Late San Nicolas	South California	T	T	33.2492	-119.5057	Scheib et al. [14]
SN-51	ancient	Late San Nicolas	South California	T	T	33.2492	-119.5057	Scheib et al. [14]
SN-52	ancient	Late San Nicolas	South California	T	T	33.2492	-119.5057	Scheib et al. [14]
CT-01	ancient	Late South Chanel Islands	South California	T	T	33.385	-118.404	Scheib et al. [14]
SC-04	ancient	Late South Chanel Islands	South California	T	T	32.9	-118.48	Scheib et al. [14]
SC-05	ancient	Late South Chanel Islands	South California	T	T	32.9	-118.48	Scheib et al. [14]
SC-1	ancient	Late South Chanel Islands	South California	T	T	32.9	-118.48	Scheib et al. [14]
SC-3	ancient	Late South Chanel Islands	South California	T	T	32.9	-118.48	Scheib et al. [14]
SC-6	ancient	Late South Chanel Islands	South California	T	T	32.9	-118.48	Scheib et al. [14]
CR-01	ancient	North Chanel Islands	South California	T	T	34.021	-119.753	Scheib et al. [14]
SM-01	ancient	North Chanel Islands	South California	T	T	34.041	-120.365	Scheib et al. [14]
SM-02	ancient	North Chanel Islands	South California	T	T	34.041	-120.365	Scheib et al. [14]
NC-C	ancient	Santa Barbara	South California	T	T	34.942	-119.688	Scheib et al. [14]
PS-03	ancient	Santa Barbara	South California	T	T	34.903	-120.67	Scheib et al. [14]
PS-06	ancient	Santa Barbara	South California	T	T	34.903	-120.67	Scheib et al. [14]
PS-07	ancient	Santa Barbara	South California	T	T	34.903	-120.67	Scheib et al. [14]
PS-09	ancient	Santa Barbara	South California	T	T	34.903	-120.67	Scheib et al. [14]
PS-18	ancient	Santa Barbara	South California	T	T	34.903	-120.67	Scheib et al. [14]
Kennewick	ancient	Ancient One	Washington Stat	T	T	46.183	-119.093	Rasmussen et al. [31]
Aleutian_2	modern	Aleutian	Alaska	F	F	52	-176.6	Raghavan et al. [30]
Athabascan_1	modern	Athabaskan	Alaska	F	F	54.233	-125.77	Raghavan et al. [30]
Athabascan_2	modern	Athabaskan	Alaska	F	F	54.233	-125.77	Raghavan et al. [30]
HG01500	modern	IBS	European	T	F			1000 Genomes [29]
HG01501	modern	IBS	European	T	F			1000 Genomes [29]
HG01503	modern	IBS	European	T	F			1000 Genomes [29]
HG01504	modern	IBS	European	T	F			1000 Genomes [29]
HG01506	modern	IBS	European	T	F			1000 Genomes [29]
HG01507	modern	IBS	European	T	F			1000 Genomes [29]
HG01509	modern	IBS	European	T	F			1000 Genomes [29]
HG01510	modern	IBS	European	T	F			1000 Genomes [29]
HG01512	modern	IBS	European	T	F			1000 Genomes [29]

HG01513	modern	IBS	European	T	F			1000 Genomes [29]
HG01515	modern	IBS	European	T	F			1000 Genomes [29]
HG01516	modern	IBS	European	T	F			1000 Genomes [29]
HG01518	modern	IBS	European	T	F			1000 Genomes [29]
HG01519	modern	IBS	European	T	F			1000 Genomes [29]
HG01521	modern	IBS	European	T	F			1000 Genomes [29]
HG01522	modern	IBS	European	T	F			1000 Genomes [29]
HG01524	modern	IBS	European	T	F			1000 Genomes [29]
HG01525	modern	IBS	European	T	F			1000 Genomes [29]
HG01527	modern	IBS	European	T	F			1000 Genomes [29]
HG01528	modern	IBS	European	T	F			1000 Genomes [29]
HG01530	modern	IBS	European	T	F			1000 Genomes [29]
HG01531	modern	IBS	European	T	F			1000 Genomes [29]
HG01536	modern	IBS	European	T	F			1000 Genomes [29]
HG01537	modern	IBS	European	T	F			1000 Genomes [29]
HG01602	modern	IBS	European	T	F			1000 Genomes [29]
Greenlander_1	modern	Inuit	Greenland	F	F	67.5	-37.9	Raghavan et al. [30]
Greenlander_2	modern	Inuit	Greenland	F	F	67.5	-37.9	Raghavan et al. [30]
HGDP00854	modern	Maya	Mexico	T	F	19	-91	Bergstrom et al. [37]
HGDP00855	modern	Maya	Mexico	T	T	19	-91	Bergstrom et al. [37]
HGDP00856	modern	Maya	Mexico	T	F	19	-91	Bergstrom et al. [37]
HGDP00857	modern	Maya	Mexico	T	T	19	-91	Bergstrom et al. [37]
HGDP00858	modern	Maya	Mexico	T	F	19	-91	Bergstrom et al. [37]
HGDP00859	modern	Maya	Mexico	T	F	19	-91	Bergstrom et al. [37]
HGDP00860	modern	Maya	Mexico	T	F	19	-91	Bergstrom et al. [37]
HGDP00861	modern	Maya	Mexico	T	F	19	-91	Bergstrom et al. [37]
HGDP00862	modern	Maya	Mexico	T	F	19	-91	Bergstrom et al. [37]
HGDP00863	modern	Maya	Mexico	T	F	19	-91	Bergstrom et al. [37]
HGDP00864	modern	Maya	Mexico	T	F	19	-91	Bergstrom et al. [37]
HGDP00865	modern	Maya	Mexico	T	F	19	-91	Bergstrom et al. [37]
HGDP00868	modern	Maya	Mexico	T	F	19	-91	Bergstrom et al. [37]
HGDP00869	modern	Maya	Mexico	T	F	19	-91	Bergstrom et al. [37]
HGDP00870	modern	Maya	Mexico	T	F	19	-91	Bergstrom et al. [37]
HGDP00871	modern	Maya	Mexico	T	F	19	-91	Bergstrom et al. [37]
HGDP00872	modern	Maya	Mexico	T	F	19	-91	Bergstrom et al. [37]
HGDP00873	modern	Maya	Mexico	T	F	19	-91	Bergstrom et al. [37]
HGDP00875	modern	Maya	Mexico	T	F	19	-91	Bergstrom et al. [37]
HGDP00876	modern	Maya	Mexico	T	F	19	-91	Bergstrom et al. [37]
HGDP00877	modern	Maya	Mexico	T	F	19	-91	Bergstrom et al. [37]
LP6005443-DNA_F11	modern	Mixe	Mexico	T	T	16.95	-96.58	Mallick et al. [32]
SS6004479	modern	Mixe	Mexico	T	T	16.95	-96.58	Mallick et al. [32]
LP6005443-DNA_G11	modern	Mixtec	Mexico	T	T	17	-97	Mallick et al. [32]
LP6005443-DNA_H11	modern	Mixtec	Mexico	T	T	17	-97	Mallick et al. [32]
NA19648	modern	MXL	Mexico	T	T	33.9472	-118.4957	1000 Genomes [29]
NA19649	modern	MXL	Mexico	T	T	33.9472	-118.4957	1000 Genomes [29]
NA19651	modern	MXL	Mexico	T	T	33.9472	-118.4957	1000 Genomes [29]
NA19652	modern	MXL	Mexico	T	T	33.9472	-118.4957	1000 Genomes [29]
NA19654	modern	MXL	Mexico	T	T	33.9472	-118.4957	1000 Genomes [29]
NA19655	modern	MXL	Mexico	T	T	33.9472	-118.4957	1000 Genomes [29]
NA19657	modern	MXL	Mexico	T	T	33.9472	-118.4957	1000 Genomes [29]
NA19658	modern	MXL	Mexico	T	T	33.9472	-118.4957	1000 Genomes [29]

NA19661	modern	MXL	Mexico	T	T	33.9472	-118.4957	1000 Genomes [29]
NA19663	modern	MXL	Mexico	T	T	33.9472	-118.4957	1000 Genomes [29]
NA19664	modern	MXL	Mexico	T	T	33.9472	-118.4957	1000 Genomes [29]
NA19669	modern	MXL	Mexico	T	T	33.9472	-118.4957	1000 Genomes [29]
NA19670	modern	MXL	Mexico	T	T	33.9472	-118.4957	1000 Genomes [29]
NA19676	modern	MXL	Mexico	T	T	33.9472	-118.4957	1000 Genomes [29]
NA19678	modern	MXL	Mexico	T	T	33.9472	-118.4957	1000 Genomes [29]
NA19679	modern	MXL	Mexico	T	T	33.9472	-118.4957	1000 Genomes [29]
NA19681	modern	MXL	Mexico	T	T	33.9472	-118.4957	1000 Genomes [29]
NA19682	modern	MXL	Mexico	T	T	33.9472	-118.4957	1000 Genomes [29]
NA19684	modern	MXL	Mexico	T	T	33.9472	-118.4957	1000 Genomes [29]
NA19716	modern	MXL	Mexico	T	T	33.9472	-118.4957	1000 Genomes [29]
NA19717	modern	MXL	Mexico	T	T	33.9472	-118.4957	1000 Genomes [29]
NA19719	modern	MXL	Mexico	T	T	33.9472	-118.4957	1000 Genomes [29]
HGDP01037	modern	Pima	Mexico	T	F	29	-108	Bergstrom et al. [37]
HGDP01043	modern	Pima	Mexico	T	F	29	-108	Bergstrom et al. [37]
HGDP01044	modern	Pima	Mexico	T	T	29	-108	Bergstrom et al. [37]
HGDP01047	modern	Pima	Mexico	T	T	29	-108	Bergstrom et al. [37]
HGDP01050	modern	Pima	Mexico	T	F	29	-108	Bergstrom et al. [37]
HGDP01053	modern	Pima	Mexico	T	F	29	-108	Bergstrom et al. [37]
HGDP01055	modern	Pima	Mexico	T	F	29	-108	Bergstrom et al. [37]
HGDP01056	modern	Pima	Mexico	T	F	29	-108	Bergstrom et al. [37]
HGDP01057	modern	Pima	Mexico	T	F	29	-108	Bergstrom et al. [37]
HGDP01058	modern	Pima	Mexico	T	F	29	-108	Bergstrom et al. [37]
HGDP01059	modern	Pima	Mexico	T	F	29	-108	Bergstrom et al. [37]
HGDP01060	modern	Pima	Mexico	T	F	29	-108	Bergstrom et al. [37]
LP6005443-DNA_A12	modern	Zapotec	Mexico	T	T	16.5	-97.2	Mallick et al. [32]
LP6005677-DNA_D01	modern	Zapotec	Mexico	T	T	16.5	-97.2	Mallick et al. [32]
HGDP01223	modern	Mongolian	Mongolian	F	F	48.5	119	Bergstrom et al. [37]
HGDP01224	modern	Mongolian	Mongolian	F	F	48.5	119	Bergstrom et al. [37]
HGDP01225	modern	Mongolian	Mongolian	F	F	48.5	119	Bergstrom et al. [37]
HGDP01227	modern	Mongolian	Mongolian	F	F	48.5	119	Bergstrom et al. [37]
HGDP01228	modern	Mongolian	Mongolian	F	F	48.5	119	Bergstrom et al. [37]
HGDP01229	modern	Mongolian	Mongolian	F	F	48.5	119	Bergstrom et al. [37]
HGDP01230	modern	Mongolian	Mongolian	F	F	48.5	119	Bergstrom et al. [37]
HGDP01231	modern	Mongolian	Mongolian	F	F	48.5	119	Bergstrom et al. [37]
HGDP01232	modern	Mongolian	Mongolian	F	F	48.5	119	Bergstrom et al. [37]
HAI001	modern	Haida	PNW	F	F	54.017	-132.15	Verdu et al. [15]
HAI002	modern	Haida	PNW	F	F	54.017	-132.15	Verdu et al. [15]
HAI003	modern	Haida	PNW	F	F	54.017	-132.15	Verdu et al. [15]
HAI004	modern	Haida	PNW	F	F	54.017	-132.15	Verdu et al. [15]
HAI005	modern	Haida	PNW	F	F	54.017	-132.15	Verdu et al. [15]
HAI006	modern	Haida	PNW	F	F	54.017	-132.15	Verdu et al. [15]
HAI007	modern	Haida	PNW	F	F	54.017	-132.15	Verdu et al. [15]
HAI008	modern	Haida	PNW	F	F	54.017	-132.15	Verdu et al. [15]
HAI009	modern	Haida	PNW	F	F	54.017	-132.15	Verdu et al. [15]
HAI010	modern	Haida	PNW	F	F	54.017	-132.15	Verdu et al. [15]
NIS001	modern	Nisga'a	PNW	F	F	50.017	-129.547	Verdu et al. [15]
NIS002	modern	Nisga'a	PNW	F	F	50.017	-129.547	Verdu et al. [15]
NIS003	modern	Nisga'a	PNW	F	F	50.017	-129.547	Verdu et al. [15]
NIS004	modern	Nisga'a	PNW	F	F	50.017	-129.547	Verdu et al. [15]

NIS005	modern	Nisga'a	PNW	F	F	50.017	-129.547	Verdu et al. [15]
NIS006	modern	Nisga'a	PNW	F	F	50.017	-129.547	Verdu et al. [15]
NIS007	modern	Nisga'a	PNW	F	F	50.017	-129.547	Verdu et al. [15]
NIS008	modern	Nisga'a	PNW	F	F	50.017	-129.547	Verdu et al. [15]
SPL001	modern	Splatsin	PNW	F	F	50.551	-119.14	Verdu et al. [15]
SPL002	modern	Splatsin	PNW	F	F	50.551	-119.14	Verdu et al. [15]
SPL003	modern	Splatsin	PNW	F	F	50.551	-119.14	Verdu et al. [15]
SPL004	modern	Splatsin	PNW	F	F	50.551	-119.14	Verdu et al. [15]
SPL005	modern	Splatsin	PNW	F	F	50.551	-119.14	Verdu et al. [15]
SPL006	modern	Splatsin	PNW	F	F	50.551	-119.14	Verdu et al. [15]
SPL007	modern	Splatsin	PNW	F	F	50.551	-119.14	Verdu et al. [15]
SPL008	modern	Splatsin	PNW	F	F	50.551	-119.14	Verdu et al. [15]
SPL009	modern	Splatsin	PNW	F	F	50.551	-119.14	Verdu et al. [15]
STS002	modern	Stswecem'c	PNW	F	F	51.593	-122.089	Verdu et al. [15]
STS003	modern	Stswecem'c	PNW	F	F	51.593	-122.089	Verdu et al. [15]
STS004	modern	Stswecem'c	PNW	F	F	51.593	-122.089	Verdu et al. [15]
STS005	modern	Stswecem'c	PNW	F	F	51.593	-122.089	Verdu et al. [15]
STS006	modern	Stswecem'c	PNW	F	F	51.593	-122.089	Verdu et al. [15]
STS007	modern	Stswecem'c	PNW	F	F	51.593	-122.089	Verdu et al. [15]
STS008	modern	Stswecem'c	PNW	F	F	51.593	-122.089	Verdu et al. [15]
STS009	modern	Stswecem'c	PNW	F	F	51.593	-122.089	Verdu et al. [15]
STS010	modern	Stswecem'c	PNW	F	F	51.593	-122.089	Verdu et al. [15]
STS011	modern	Stswecem'c	PNW	F	F	51.593	-122.089	Verdu et al. [15]
STS012	modern	Stswecem'c	PNW	F	F	51.593	-122.089	Verdu et al. [15]
STS013	modern	Stswecem'c	PNW	F	F	51.593	-122.089	Verdu et al. [15]
TLI001	modern	Tlingit	PNW	F	F	57.052	-135.339	Verdu et al. [15]
TLI002	modern	Tlingit	PNW	F	F	57.052	-135.339	Verdu et al. [15]
TLI003	modern	Tlingit	PNW	F	F	57.052	-135.339	Verdu et al. [15]
TLI004	modern	Tlingit	PNW	F	F	57.052	-135.339	Verdu et al. [15]
TLI005	modern	Tlingit	PNW	F	F	57.052	-135.339	Verdu et al. [15]
TLI006	modern	Tlingit	PNW	F	F	57.052	-135.339	Verdu et al. [15]
TLI007	modern	Tlingit	PNW	F	F	57.052	-135.339	Verdu et al. [15]
TLI008	modern	Tlingit	PNW	F	F	57.052	-135.339	Verdu et al. [15]
TLI009	modern	Tlingit	PNW	F	F	57.052	-135.339	Verdu et al. [15]
TLI010	modern	Tlingit	PNW	F	F	57.052	-135.339	Verdu et al. [15]
TLI011	modern	Tlingit	PNW	F	F	57.052	-135.339	Verdu et al. [15]
TLI012	modern	Tlingit	PNW	F	F	57.052	-135.339	Verdu et al. [15]
TLI013	modern	Tlingit	PNW	F	F	57.052	-135.339	Verdu et al. [15]
TLI014	modern	Tlingit	PNW	F	F	57.052	-135.339	Verdu et al. [15]
TLI015	modern	Tlingit	PNW	F	F	57.052	-135.339	Verdu et al. [15]
TLI016	modern	Tlingit	PNW	F	F	57.052	-135.339	Verdu et al. [15]
TSI001	modern	Tsimshian	PNW	F	F	54.442	-130.433	Verdu et al. [15]
TSI003	modern	Tsimshian	PNW	F	F	54.442	-130.433	Verdu et al. [15]
TSI004	modern	Tsimshian	PNW	F	F	54.442	-130.433	Verdu et al. [15]
TSI005	modern	Tsimshian	PNW	F	F	54.442	-130.433	Verdu et al. [15]
TSI006	modern	Tsimshian	PNW	F	F	54.442	-130.433	Verdu et al. [15]
TSI007	modern	Tsimshian	PNW	F	F	54.442	-130.433	Verdu et al. [15]
TSI008	modern	Tsimshian	PNW	F	F	54.442	-130.433	Verdu et al. [15]
TSI009	modern	Tsimshian	PNW	F	F	54.442	-130.433	Verdu et al. [15]
TSI010	modern	Tsimshian	PNW	F	F	54.442	-130.433	Verdu et al. [15]
TSI011	modern	Tsimshian	PNW	F	F	54.442	-130.433	Verdu et al. [15]

TSI012	modern	Tsimshian	PNW	F	F	54.442	-130.433	Verdu et al. [15]
TSI013	modern	Tsimshian	PNW	F	F	54.442	-130.433	Verdu et al. [15]
TSI014	modern	Tsimshian	PNW	F	F	54.442	-130.433	Verdu et al. [15]
TSI015	modern	Tsimshian	PNW	F	F	54.442	-130.433	Verdu et al. [15]
TSI016	modern	Tsimshian	PNW	F	F	54.442	-130.433	Verdu et al. [15]
TSI017	modern	Tsimshian	PNW	F	F	54.442	-130.433	Verdu et al. [15]
TSI018	modern	Tsimshian	PNW	F	F	54.442	-130.433	Verdu et al. [15]
TSI019	modern	Tsimshian	PNW	F	F	54.442	-130.433	Verdu et al. [15]
TSI021	modern	Tsimshian	PNW	F	F	54.442	-130.433	Verdu et al. [15]
TSI022	modern	Tsimshian	PNW	F	F	54.442	-130.433	Verdu et al. [15]
TSI023	modern	Tsimshian	PNW	F	F	54.442	-130.433	Verdu et al. [15]
TSI024	modern	Tsimshian	PNW	F	F	54.442	-130.433	Verdu et al. [15]
TSI025	modern	Tsimshian	PNW	F	F	54.442	-130.433	Verdu et al. [15]
TSI026	modern	Tsimshian	PNW	F	F	54.442	-130.433	Verdu et al. [15]
M01	modern	Muwekma Ohlone	SF Bay Area	T	T	37.534	-121.885	*This study*
M02	modern	Muwekma Ohlone	SF Bay Area	T	T	37.534	-121.885	*This study*
M03	modern	Muwekma Ohlone	SF Bay Area	T	T	37.534	-121.885	*This study*
M04	modern	Muwekma Ohlone	SF Bay Area	T	T	37.534	-121.885	*This study*
M05	modern	Muwekma Ohlone	SF Bay Area	T	T	37.534	-121.885	*This study*
M06	modern	Muwekma Ohlone	SF Bay Area	T	T	37.534	-121.885	*This study*
M07	modern	Muwekma Ohlone	SF Bay Area	T	T	37.534	-121.885	*This study*
M08	modern	Muwekma Ohlone	SF Bay Area	T	T	37.534	-121.885	*This study*
Alt1	modern	Altai	Siberia	F	F	56.3	82.8	Raghavan et al. [30]
Alt2	modern	Altai	Siberia	F	F	56.3	82.8	Raghavan et al. [30]
LP6005443-DNA_D03	modern	Chaplin	Siberia	F	F	64.48	172.86	Mallick et al. [32]
LP6005443-DNA_C03	modern	Chukchi	Siberia	F	F	69	169	Mallick et al. [32]
LP6005443-DNA_D04	modern	Itelman	Siberia	F	F	57	157	Mallick et al. [32]
Ket1	modern	Ket	Siberia	F	F	63.8	87.4	Raghavan et al. [30]
Ket2	modern	Ket	Siberia	F	F	63.8	87.4	Raghavan et al. [30]
LP6005443-DNA_F03	modern	Naukan	Siberia	F	F	66.02	169.71	Mallick et al. [32]
LP6005443-DNA_G03	modern	Naukan	Siberia	F	F	66.02	169.71	Mallick et al. [32]
Nivh1	modern	Nivkh	Siberia	F	F	54.5	136.5	Raghavan et al. [30]
Nivh2	modern	Nivkh	Siberia	F	F	54.5	136.5	Raghavan et al. [30]
Yak1	modern	Sakha	Siberia	F	F	63	130	Raghavan et al. [30]
Yak2	modern	Sakha	Siberia	F	F	63	130	Raghavan et al. [30]
LP6005443-DNA_B03	modern	Sireniki	Siberia	F	F	64.4	173.9	Mallick et al. [32]
LP6005443-DNA_H03	modern	Sireniki	Siberia	F	F	64.4	173.9	Mallick et al. [32]
Esk17	modern	Yupik	Siberia	F	F	64.41	-172.267	Raghavan et al. [30]
Esk20	modern	Yupik	Siberia	F	F	64.41	-172.267	Raghavan et al. [30]
LP6005519-DNA_D01	modern	Chane	South America	F	T	-22.53	-63.82	Mallick et al. [32]
HGDP00702	modern	Colombian	South America	T	T	3	-68	Bergstrom et al. [37]
HGDP00703	modern	Colombian	South America	T	F	3	-68	Bergstrom et al. [37]
HGDP00704	modern	Colombian	South America	T	F	3	-68	Bergstrom et al. [37]
HGDP00970	modern	Colombian	South America	T	F	3	-68	Bergstrom et al. [37]
HGDP00995	modern	Karitiana	South America	F	F	-10	-63	Bergstrom et al. [37]
HGDP00999	modern	Karitiana	South America	F	F	-10	-63	Bergstrom et al. [37]
HGDP01001	modern	Karitiana	South America	F	F	-10	-63	Bergstrom et al. [37]
HGDP01009	modern	Karitiana	South America	F	F	-10	-63	Bergstrom et al. [37]
HGDP01010	modern	Karitiana	South America	F	F	-10	-63	Bergstrom et al. [37]
HGDP01012	modern	Karitiana	South America	F	T	-10	-63	Bergstrom et al. [37]
HGDP01013	modern	Karitiana	South America	F	F	-10	-63	Bergstrom et al. [37]

HGDP01014	modern	Karitiana	South America	F	F	-10	-63	Bergstrom et al. [37]
HGDP01015	modern	Karitiana	South America	F	T	-10	-63	Bergstrom et al. [37]
HGDP01018	modern	Karitiana	South America	F	T	-10	-63	Bergstrom et al. [37]
HGDP01019	modern	Karitiana	South America	F	F	-10	-63	Bergstrom et al. [37]
LP6005519-DNA_G02	modern	Quechua	South America	F	T	-13.5	-72	Mallick et al. [32]
LP6005677-DNA_E01	modern	Quechua	South America	F	T	-13.5	-72	Mallick et al. [32]
LP6005677-DNA_F01	modern	Quechua	South America	F	T	-13.5	-72	Mallick et al. [32]
HGDP00832	modern	Surui	South America	F	F	-11	-62	Bergstrom et al. [37]
HGDP00837	modern	Surui	South America	F	F	-11	-62	Bergstrom et al. [37]
HGDP00838	modern	Surui	South America	F	F	-11	-62	Bergstrom et al. [37]
HGDP00843	modern	Surui	South America	F	F	-11	-62	Bergstrom et al. [37]
HGDP00845	modern	Surui	South America	F	F	-11	-62	Bergstrom et al. [37]
HGDP00846	modern	Surui	South America	F	T	-11	-62	Bergstrom et al. [37]
HGDP00849	modern	Surui	South America	F	F	-11	-62	Bergstrom et al. [37]
HGDP00852	modern	Surui	South America	F	T	-11	-62	Bergstrom et al. [37]

Table S5: f_4 tests comparing similarities of populations. Each row corresponds to a test in which the similarity of populations 2 and 3 is compared to the similarity of populations 1 and 3. A negative value for the f_4 statistic indicates greater similarity of populations 2 and 3. The increasing p-values for the first three rows indicate that evidence of similarity is stronger between Muwekma Ohlone and Rummey Ta Kuččuwiš Tiprectak and Síi Túupentak than between Muwekma Ohlone and North Channel Islands and Santa Barbara, whose similarity is in turn stronger than that between Muwekma Ohlone and South Channel islands and San Nicolas. In the bottom two rows, we again see that evidence of similarity between Muwekma Ohlone and Rummey Ta Kuččuwiš Tiprectak and Síi Túupentak is stronger than that between Muwekma Ohlone and North Channel Islands and Santa Barbara, which is in turn stronger than that between Muwekma Ohlone and South Channel Islands and San Nicolas. The table shows all f_4 tests that we performed.

**Population 1**	**Population 2**	**Population 3**	**Population 4**	***f*4 statistic**	**z-score**	**p-value**
Neo-Aleutian	Rummey Ta Kuččuwiš Tiprectak and Síi Túupentak	Muwekma Ohlone	IBS	-5.46×10^{-3}	-28.7	1.22×10^{-180}
Neo-Aleutian	North Channel Islands and Santa Barbara	Muwekma Ohlone	IBS	-4.97×10^{-3}	-24.8	3.84×10^{-136}
Neo-Aleutian	South Channel Islands and San Nicolas	Muwekma Ohlone	IBS	-4.36×10^{-3}	-23.5	1.16×10^{-121}
North Channel Islands and Santa Barbara	Rummey Ta Kuččuwiš Tiprectak and Síi Túupentak	Muwekma Ohlone	IBS	-6.27×10^{-4}	-4.39	1.11×10^{-5}
South Channel Islands and San Nicolas	Rummey Ta Kuččuwiš Tiprectak and Síi Túupentak	Muwekma Ohlone	IBS	-1.30×10^{-3}	-9.59	8.41×10^{-22}
